# The record of Aplodontidae (Rodentia, Mammalia) in the Oligocene and Miocene of the Valley of Lakes (Central Mongolia) with some comments on the morphologic variability

**DOI:** 10.1007/s12549-016-0255-y

**Published:** 2016-11-26

**Authors:** Olivier Maridet, Gudrun Daxner-Höck, Paloma López-Guerrero, Ursula B. Göhlich

**Affiliations:** 1 JURASSICA Museum, route de Fontenais 21, 2900 Porrentruy, Switzerland; 2 Department of Geosciences, Earth Sciences, University of Fribourg, Chemin du Musée 6, Pérolles, 1700 Fribourg, Switzerland; 3 Rupertusstr. 16, 5201 Seekirchen, Austria; 4 Geologisch-Paläontologische Abt., Naturhistorisches Museum Wien, Burgring 7 A, 1010 Vienna, Austria; 5 Departamento de Paleontología, Facultad de Ciencias Geológicas, Universidad Complutense de Madrid, C/ José Antonio Novais, 2, 28040 Madrid, Spain

**Keywords:** Mongolia, Oligocene, Miocene, Rodentia, Systematics

## Abstract

The present publication reports new discoveries of Oligocene and early Miocene aplodontid rodents from the Taatsiin Gol area (Valley of Lakes) in Mongolia. The fossil aplodontids recovered in this area are mainly composed of dental remains, some fragmentary jaws plus one partially preserved skull. Aplodontid rodents have been found from the early Oligocene (local biozone A) to the early middle Miocene (local biozone D). Altogether, eight taxa belonging to five genera have been identified in the investigated deposits: *Ninamys arboraptus*, *Ninamys kazimierzi*, *Promeniscomys* cf. *sinensis*, *Prosciurus*? *mongoliensis* and *Prosciurus*? sp. nov. in the early Oligocene; *N. arboraptus*, *Proansomys badamae* sp. nov. and Ansomyinae indet. in the late Oligocene; and *Ansomys* sp.1 in the early Miocene. In addition, although outside of the topic of the present special issue, one additional taxon, *Ansomys* sp.2, is reported from the ?middle Miocene. The material of aplodontids is usually relatively scarce in Asian localities. For the first time, with a sample size of 81 specimens, the material from Central Mongolia (mainly from the Oligocene) now allows a more accurate description of the morphological and size variability and resolves some systematic problems. The study of these aplodontids reveals that they are more abundant and diverse in the early Oligocene and that the diversity decreases during the late Oligocene and Miocene. One hypothesis, to explain the opposite diversity trend observed previously for sciurids in the same region, is that both Sciuromorpha families might have competed for the same resources from the early Oligocene to the middle Miocene in Central Mongolia.

**Electronic supplementary material:**

The online version of this article (doi:10.1007/s12549-016-0255-y) contains supplementary material, which is available to authorized users.

## Introduction

Since 1995, three successive joint Austrian-Mongolian projects have been carried out in the Taatsiin Gol area in Mongolia (see Daxner-Höck et al. 2017, fig. 3, for an introduction and a map with the location of each locality), including eight field missions. During these missions, an extensive sampling has been undertaken, comprised of not only surface sampling but also screen-washing of almost a hundred tons of sediment from more than 40 localities and sections spanning a time range from the early Oligocene to the late Miocene. This continental sequence has allowed a precise stratigraphic adjustment based on the evolution of mammals and age determination of basalts as elaborated by Daxner-Höck et al. (1997) and Höck et al. (1999). A part of the mammalian fossil record has already been studied in detail including the Ruminantia (Vislobokova and Daxner-Höck 2002); Rhinocerotidae (Heissig 2007); Proboscidea (Göhlich 2007); Marsupialia, Erinaceomorpha and Soricomorpha (Ziegler et al. 2007); and most of Rodentia (e.g. Daxner-Höck 2000, 2001; Daxner-Höck and Wu 2003; Schmidt-Kittler et al. 2007; Daxner-Höck et al. 2014, 2015; Maridet et al. 2014a, b, 2015; Wessels et al. 2014; López-Guerrero et al. 2015). Moreover, preliminary results are available for Didymoconidae, Creodonta and Carnivora (e.g. Nagel and Morlo 2003; Morlo and Nagel 2006, 2007) and Lagomorpha (e.g. Erbajeva 2007; Erbajeva and Daxner-Höck 2014).

In this publication, we carry on the study of rodents by presenting a detailed description of the aplodontid rodents from diverse layers of early Oligocene to middle? Miocene age.

This material is mainly composed of teeth, but fragmentary jaws and one partly preserved skull have also been found. Altogether the material comprises 81 specimens, which is noticeably scarce compared to other rodent groups found in the same region (e.g. more than 3000 dipodid rodents in Daxner-Höck et al. 2014). However, considering that this family is very rare in the Oligocene and Miocene fossil record of Asia, the specimens described below also represent an important assemblage compared to other Northern Asian localities (northern China, Kazakhstan).

Recent studies focussing on Aplodontids proposed phylogenies (Hopkins 2008; Bi et al. 2013; Vianey-Liaud et al. 2013). However, the systematic palaeontology of Asian taxa remains poorly understood. This is due to the fact that genera and species have sometimes been described on the basis of a few isolated specimens and that a part of their morphology is unknown (Table 1). The new specimens described below now allow to more accurately qualify the morphological and size variability and to tackle some systematic problems.

**Table 1:** List of aplodontid taxa known in from the early Oligocene to the middle Miocene in Northern Asia and their known dentition elements

Genus/species	Authors	dp4	p4	m1/2	m3	P3	DP4	P4	M1/2	M3	References
*Prosciurus*? *mongoliensis* ^a^	Wang and Dashzeveg, 2005		X	X	X						Wang and Dashzeveg (2005)
*Prosciurus*? nov. sp.^a^	This study		X	X	X						=*Prosciurus* cf. *mongoliensis* sensu Wang and Dashzeveg (2005)
*Prosciurus*? *ordosicus*	Wang, 1987								X		Wang (1987)
*Prosciurus*? *pisinnus*	Wang and Dashzeveg, 2005		X	X	X						Wang and Dashzeveg (2005)
*Promeniscomys sinensis*	Wang, 1987					X		X	X		Wang (1987), Vianey-Liaud et al. (2013)
*Promeniscomys* cf. *sinensis* ^a^	This study		**N**	**N**	**N**			**N**	**N**	**N**	–
*Proansomys dureensis*	Bi, 2013	X	X	X	X		X	X	X	X	Bi et al. (2013)
*Proansomys daxnerae*	(Lopatin, 2000)			X							Lopatin (2000, 2004)
*Proansomys badamae* sp. nov.^a^	This study		**N**	**N**	**N**			**N**	**N**	**N**	–
*Ninamys arboraptus* ^a^	(Shevyreva, 1966)		X	X	X		**N**	X	X	**N**	Kowalski (1974), Wang (1987), Vianey-Liaud et al. (2013)
*Ninamys kazimierzi* ^a^	Vianey-Liaud, 2013	**N**	X	X	X	X		X	X	**N**	Vianey-Liaud et al. (2013)
*Ansomyinae* indet.^a^	This study		**N**	**N**	**N**						–
*Ansomys crucifer*	Lopatin, 1997		X								Lopatin (1997)
*Ansomys orientalis*	Qiu, 1987	X	X	X	X		X	X	X	X	Qiu (1987)
*Ansomys shantungensis*	(Rensberger and Li, 1986)			X							Rensberger and Li (1986)
*Ansomys shanwangensis*	Qiu and Sun, 1988		X	X	X	X	X	X	X	X	Qiu and Sun (1988)
*Ansomys* sp.1^a^	This study								**N**		–
*Ansomys* sp.2^a^	This study			**N**					X		=*Ansomys* sp. sensu Qiu (1996)

*X* previously known elements of the dentition, *N* new elements of the dentition described in the present study

^a^Taxa found in the fossil record of the Valley of Lakes

## Materials and methods

For the present investigation, specimens are stored in the collections of the Geological–Paleontological Department, Museum of Natural History in Vienna, Austria (NHMW). They are catalogued under the following numbers: NHMW 2009/0137/0001 and NHMW 2009/0138/0001 (already published by Daxner-Höck et al. 2010), then from NHMW 2015/0345/0001 to NHMW 2015/0385/0003. A detailed list of the specimens with their measurements is given in the electronic supplementary material (ESM).

**Fig. 1:**
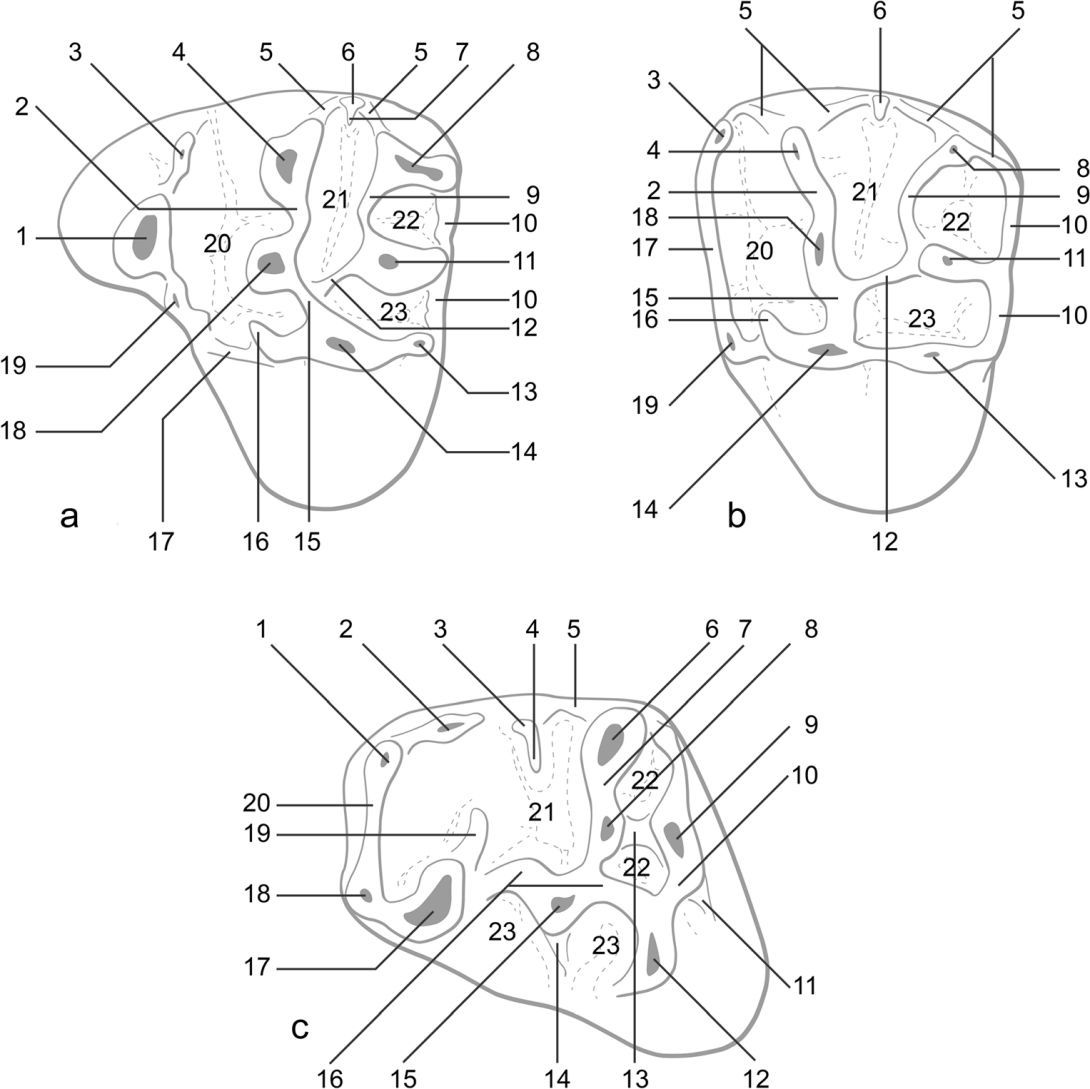
The terminology used to describe the molars modified after Wang (1987) and Vianey-Liaud et al. (2013). Sketch drawing of upper (**a** P4; **b** M1/2) and lower (**c** m1/2) cheek teeth and their terminology. Upper cheek teeth: *1*, anterocone; *2*, labial protoloph; *3*, parastyle; *4*, paracone; *5*, ectoloph; *6*, mesostyle; *7*, mesoloph; *8*, metacone; *9*, labial metaloph; *10*, posteroloph; *11*, metaconule; *12*, lingual metaloph; *13*, hypocone; *14*, protocone; *15*, lingual protoloph; *16*, anterior arm of the protocone; *17*, anteroloph; *18*, paraconule (=protoconule sensu Wang 1987); *19*, anterostyle; *20*, anterior sinus; *21*, central sinus; *22*, postero-labial sinus; *23*, postero-lingual sinus. Lower cheek teeth: *1*, metaconid; *2*, metastylid crest; *3*, mesostylid; *4*, mesolophid; *5*, entoconid ridge; *6*, entoconid; *7*, entolophid; *8*, entoconulid; *9*, hypoconulid; *10*, lingual posterolophid; *11*, labial posterolophid; *12*, hypoconid; *13*, entoconulid crest; *14*, ectomesolophid; *15*, mesoconid; *16*, ectolophid; *17*, protoconid; *18*, anteroconulid; *19*, metalophid II; *20*, metalophid I; *21*, talonid basin; *22*, posterior sinusid; *23*, sinusid

Observations and measurements were done with a binocular microscope Leica WILD M8 allowing precision to 0.01 mm. The terminology used to describe the molars is presented in Fig. 1 and modified after Wang (1987) and Vianey-Liaud et al. (2013). A clear distinction between the first and second molars was not always possible on isolated teeth; as a consequence, these tooth positions are not always separated and are described as M1/2 and m1/2. All the measurements are given in millimetres.

### Abbreviations

*Localities*. DEL = Del; HL = Khongil; IKH = Ikh Argalatyn Nuruu; RHN = Huch Teeg; SHG = Hsanda Gol; TAT = Tatal Gol; TGL = Taatsiin Gol left; TGR = Taatsiin Gol right; TGW = Toglorhoi; UNCH = Unkheltseg; UTO = Ulaan Tolgoi (see Daxner-Höck et al. 2017, fig. 3, for a map with the location of each locality).

*Teeth*. DP, dp = deciduous premolar; P, p = premolar; M, m = molar. Upper case letters indicate upper teeth, whereas lower case letters indicate lower teeth.

*Measurements*. L = maximal length; W = maximal width; TrW = trigonid width; TaW = Talonid width.

*Institution*. NHMW = Naturhistorisches Museum Wien (Austria)

### Geologic and chronologic framework

All the described specimens have been collected in the area of the “Valley of Lakes” in Central Mongolia. This area is part of the Pre-Altai depressions which are situated in western and south-central Mongolia, between the Mongolian Altai and the Gobi Altai Mountains in the south and the Khangai Mountains in the north. Here, above the Proterozoic and Palaeozoic basement, continental sediments are deposited ranging continuously from the Cretaceous to the Quaternary. A succession of four geologic formations is recognized in this region from the late Eocene to the late Miocene: Tsagaan Ovoo, Hsanda Gol, Loh and Tuyn Gol [see Daxner-Höck and Badamgarav (2007) and Daxner-Höck et al. (2017) for a detailed presentation of the geologic and chronologic framework]. In this area, the Cenozoic sedimentary sequence interfingers with several basalt deposits: three coherent basaltic layers (basalt I, II, III) of variable thickness were distinguished and dated by the 40Ar/39Ar method (Daxner-Höck et al. 1997; Höck et al. 1999). Basalt I erupted around 31.5 Ma (range 30.4–32.1) (early Oligocene), basalt II is about 28.0 Ma (range 27.0–28.0) (late Oligocene) and basalt III is about 13 Ma (range 12.2–13.2) (middle Miocene). Basalt II is mainly exposed in the very north of the mapped area near the Unzing Khurem area and the Olon Ovoony Khurem area; its thickness also strongly varies: 5–7 m in the Unzing Khurem area, whereas it can exceed 25 m in the Olon Ovoony Khurem area (Daxner-Höck and Badamgarav 2007). The succession of rodent assemblages yielded by the different localities can be grouped into eight informal, local biozones: A, B, C, C1, D, D1/1, D1/2 and E. Each informal biozone was defined by its lithostratigraphic position and the characteristic rodents from all respective fossil horizons (see Daxner-Höck and Badamgarav 2007 and Daxner-Höck et al. 2010). The present study focusses on the aplodontid material from the faunal assemblages A and B (early Oligocene), C and C1 (late Oligocene) and D1/2 (middle? Miocene).

## Systematic palaeontology

Order Rodentia Bowdich, 1821Suborder Sciuromorpha Brandt, 1855Superfamily Aplodontoidea Trouessart, 1897Family Aplodontidae Brandt, 1855Subfamily indet.Genus *Ninamys* Vianey-Liaud et al., 2013

*Ninamys arboraptus* (Shevyreva, 1966)

Fig. 2a–h

**Fig. 2:**
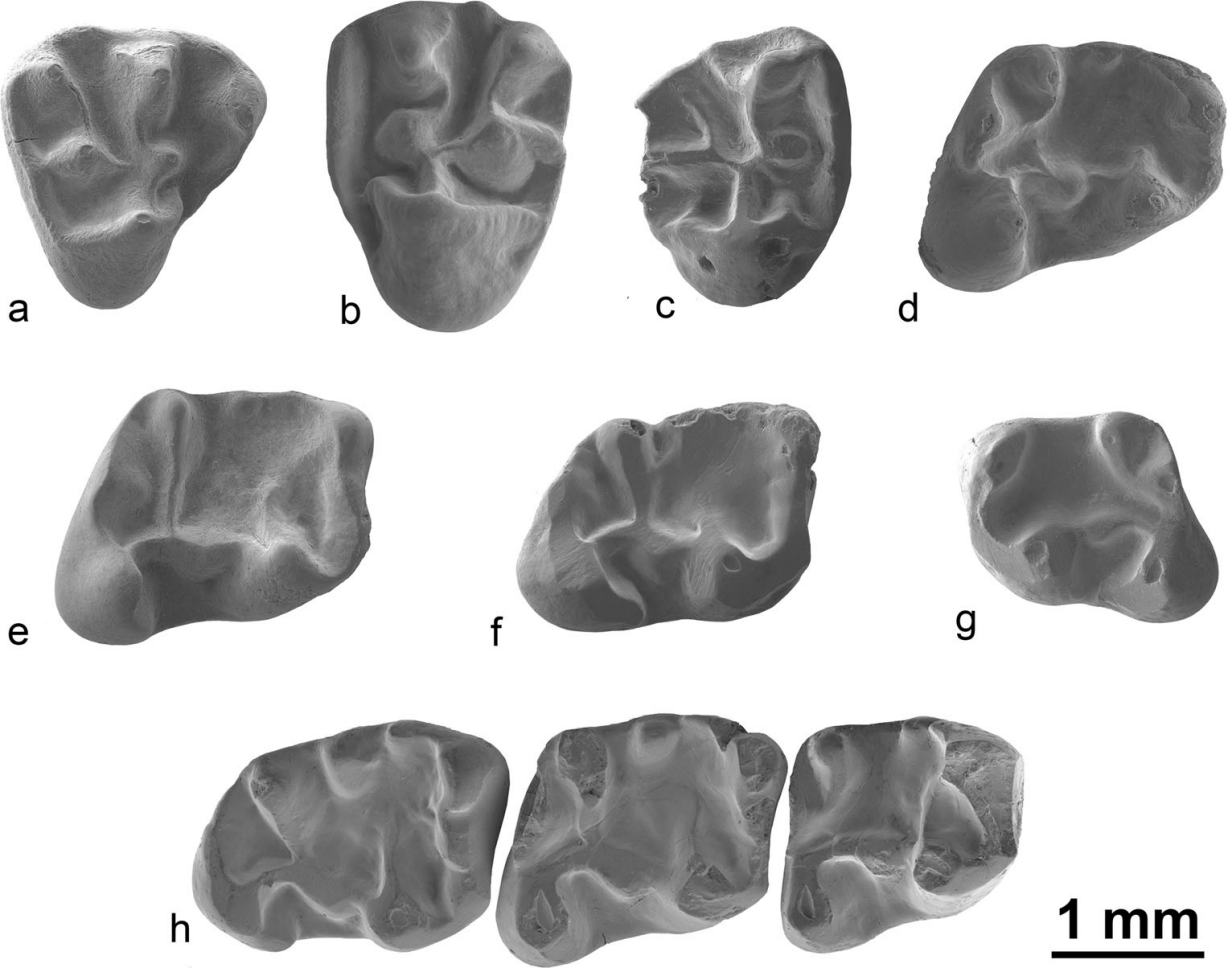
*Ninamys arboraptus* from the early Oligocene localities Taatsiin Gol right (*TGR*), Unkheltseg (*UNCH*), Ikh Argalatyn Nuruu (*IKH*) and Hsanda Gol (*SHG*), local biozones A and B, occlusal view: **a** right DP4, TGR-AB/21 (NHMW 2015/0364/0001); **b** left M1/2, UNCH-A/3 (NHMW 2015/0369/0001); **c** right M3, SHG-C/2 (NHMW 2015/0347/0001); **d** right m1, IKH-A/1 + 2 (NHMW 2015/0357/0004); **e** right m2, TGR-B/1 (NHMW 2015/0367/0003); **f** right m3, TGR-B/1 (NHMW 2015/0362/0001); **g** left m1, IKH-A/1 + 2 (NHMW 2015/0357/0002); **h** right m1-m3, TGR B/1 (NHMW 2009/0138/0001)

### Synonymy

2010 *Prosciurus* sp.2—Daxner-Höck et al. fig. 4.12, p. 356

**Localities/stratigraphy:** Khongil (layer HL-A/1, biozone A; early Oligocene), Hsanda Gol (layers SHG-C/1 and SHG-C/2, biozone A; early Oligocene); Ikh Argalatyn Nuruu (layer IKH-A/1-2, biozone B; early Oligocene); Taatsiin Gol right (layers TGR B/1, TGR-AB/21 and TGR-B/1, biozone B; early Oligocene); Unkheltseg (layer UNCH-A/3, biozone B; early Oligocene); Toglorhoi (layer TGW-A/1, biozone C; early late Oligocene).

**Material:** 9 isolated upper molars, 10 isolated lower molars and one lower tooth row with m1-m3; see appendix (ESM) for details.

**Measurements:** Given in Table 2, see appendix (ESM) for details.

**Table 2:** Measurements (in mm) of *Ninamys arboraptus* from the localities: Khongil (HL), Hsanda Gol (SHG) and Tatal Gol (TAT), local biozone A; Ikh Argalatyn Nuruu (IKH), Taatsiin Gol left (TGL), Unkheltseg (UNCH) and Taatsiin Gol UNCH (TGR), local biozone B; Toglorhoi (TGW), local biozone C

	L						W/TaW						TrW					
	*N*	Min	Max	Mean	*σ*	CV	*N*	Min	Max	Mean	*σ*	CV	*N*	Min	Max	Mean	*σ*	CV
Biozone A																		
M1/2	2	1.69	1.70	1.70	–	–	2	2.22	2.33	2.28	–	–	–	–	–	–	–	–
M3	1	–	–	1.53	–	–	1	–	–	1.74	–	–	–	–	–	–	–	–
p4	1	–	–	1.58	–	–	1	–	–	1.49	–	–	1	–	–	1.10	–	–
Biozone B																		
DP4	1	–	–	1.74	–	–	1	–	–	1.82	–	–	–	–	–	–	–	–
P4	1	–	–	1.88	–	–	1	–	–	2.02	–	–	–	–	–	–	–	–
M1/2	2	1.58	1.60	1.59	–	–	2	2.16	2.29	2.23	–	–	–	–	–	–	–	–
M3	2	1.58	1.60	1.59	–	–	2	1.74	1.81	1.78	–	–	–	–	–	–	–	–
m1/2	7	1.70	2.11	1.83	0.15	8.15	7	1.35	1.73	1.60	0.15	9.31	7	1.21	1.52	1.38	0.11	8.07
m3	2	2.01	2.19	2.10	–	–	2	1.40	1.40	1.40	–	–	2	1.47	1.60	1.53	–	–
Biozone C																		
m1	1	–	–	1.64	–	–	1	–	–	1.57	–	–	1	–	–	1.35	–	–

*N* number of teeth measured, *Min* minimum, *Max* maximum, *σ* standard deviation, *CV* variation coefficient of Simpson

### Description

**DP4:** The tooth has a subtriangular shape. Anterocone and anterostyle are well-developed but the parastyle is absent; a labial anteroloph connects the anterocone to the paracone, the ectoloph is limited to the paracone; the paracone is larger than the metacone; the lingual metaloph connects on the paraconule; the central sinus is closed labially by a mesotyle. No roots are preserved.

**P4:** The anterior lobe is short and narrow. Anterocone and anterostyle are well-developed but the parastyle is absent, a labial anteroloph connects the anterocone to the paracone. The ectoloph is complete between the paracone and the metacone. The anterior arm of the protocone is absent. The labial protoloph is curved between the paracone and the paraconule. The lingual metaloph connects to the lingual protoloph. The tooth has three roots.

**M1/2:** The ectoloph can be weakly developed or absent on the metacone, whereas it is always well-developed on the paracone. In addition to the ectoloph, a mesostyle is also sometimes present. The hypocone is either absent or weakly developed. The labial protoloph is curved between the paracone and the paraconule. Both the paracone and metaconule are larger than metacone; the metaconule is also antero-posteriorly elongated. Anterior arm of the protocone is present but weak. Anteroloph is usually enlarged labially which could be interpreted as a weakly developed parastyle. The lingual metaloph connects to the lingual part of the paraconule or to the lingual protoloph. The tooth has three roots.

**M3:** The central sinus is closed by a strong mesostyle bearing a well-developed mesoloph. The ectoloph can be weakly developed or absent on the metacone, whereas it is always well-developed on the paracone. Both the paracone and metaconule are larger than the metacone, the metacone being much reduced. The metaconule is also antero-posteriorly elongated. The hypocone is either absent or weakly developed. The anterior arm of the protocone is present but short. The lingual metaloph connects either to the paraconule or the lingual protoloph. The tooth has three roots.

**p4:** The protoconid and the metaconid are connected by the metalophid II, the metalophid I is absent. The entolophid is curved and connects the entoconid to the hypoconulid. The hypoconid displays a ridge oriented forward but not long enough to close the sinusid. The mesoconid is well-developed with a short ectomesolophid. The talonid basin is almost closed by a cingulum. The posterior sinusid is lingually open. The hypoconulid is larger than the entoconid. A very weakly developed labial posterolophid is present. The tooth has two roots.

**m1/2:** The protoconid and the metaconid are always connected by the metalophid I, the metalophid II shows more variability (absent, incomplete or complete) and the anteroconulid is either weakly developed or absent. The entolophid is either curved and connects the entoconid to the hypoconulid (usually in m1), but it can also be straight and connect the entoconid to the ectolophid (usually in m2, see NHMW 2009/0138/0001). The hypoconid can display a ridge oriented forward but usually not long enough to close the sinusid. However, in one tooth, it is longer and it almost reaches the ectomesolophid (NHMW 2015/0357/0003). The mesoconid is either weakly developed or large with a short ectomesolophid. The talonid basin is closed (or partially closed), either by a cingulum or by a more or less developed mesotylid. The posterior sinusid is often closed by a cingulum joining the hypoconulid to the entoconid but can also be lingually open. The tooth has two roots.

**m3:** Slightly longer than the m2. The protoconid and the metaconid are connected by the metalophid I, the metalophid II is incomplete; the anteroconulid can be absent or well-developed. The entolophid is often curved and connects the entoconid to the hypoconulid, but it can also be straight and connects the entoconid to the ectolophid. The hypoconid displays a ridge oriented forward but not long enough to close the sinusid. The mesoconid is well-developed but without ectomesolophid. The talonid basin is closed by well-developed cingulum and mesotylid. The posterior sinusid is closed by a cingulum. The tooth has two roots.

**Comparisons and discussion:** The species was firstly described under the genus *Prosciurus* by Shevyreva in 1971. Later was included in the genus *Ninamys* by Vianey-Liaud et al. in 2013. The morphology of the specimens found in Mongolia generally fits the diagnosis of genus and the species as given by Vianey-Liaud et al. (2013) but also displays more variability. It is indeed noteworthy that generic diagnosis is based on few specimens. The specimens described above suggest that a revision of the generic diagnosis will probably be needed when more material is discovered, especially, concerning the variability of the ectoloph on the upper molars of *Ninamys*. For *N. arboraptus*, the shape of the protoloph, the development of the hypocone, the anterior arm of the protocone and the mesostyle on the upper molars have more variability than described by Vianey-Liaud et al. (2013). Likewise, the development of the metalophid II, the mesostylid, mesoconid and posterolophid on the lower molars also display more variability.

Concerning the morphology of the M3, only one tooth has been assigned to *N. arboraptus* from Central Asia so far (from Kazakhstan, Shevyreva 1971; illustrated by Vianey-Liaud et al. 2013, fig. 4D). But none of the M3s found in Mongolia display similar morphology. Based on their size and morphology similar to the M1/2, three M3s have been referred to *N. arboraptus* among the Mongolian material. They differ noticeably from the tooth initially included in the type population of *Prosciurus arboraptus* by Shevyreva (1971) in having well-developed paraconule, a large and antero-posteriorly elongated metaconule, a strong mesotyle with mesoloph and a posterior border less rounded. These characters are otherwise similar to those of the M1/2 which supports our referral to the same species. It is so far not possible to state whether the M3 published by Shevyreva (1971) illustrates a different morphotype included in the intraspecific variability or if it belongs to a different taxon. It is, nevertheless, worth remembering that the morphology of the M3s of *Prosciurus mongoliensis* Wang and Dashzeveg, 2005, *Prosciurus pisinnus* Wang and Dashzeveg, 2005 and *Promeniscomys sinensis* (Wang, 1987) remains so far unknown.

Wang (1987) described a new species, *Prosciurus ordosicus* based on a single M1/2. Vianey-Liaud et al. (2013) notice that the Asian Oligocene taxon assigned to *Prosciurus*, *Prosciurus ordosicus*, does not really fit the original diagnosis of the genus given by Wood (1937), nor their emended diagnosis, and questioned the generic attribution of *Prosciurus ordosicus*. The M1/2 described by Wang (1987) shows an ectoloph on the paracone but not on the metacone. Therefore, the central sinus is open labially. This trait is a particular characteristic and is now included in the morphological variability of the genus *Ninamys*. The same M1/2 also displays a metaconule stronger than the metacone and a metaloph joining the protoloph between the paraconule and the protocone. This trait is characteristic of the species *N. arboraptus*. Finally, it is noteworthy that the size of the species described by Wang (1987) is now included in the size range of *N. arboraptus* based on the new specimens presented above. The only morphological difference seems to be the straight protoloph. However, based on the material collected in the Valley of Lakes, this character seems to present a noticeable intraspecific variability too. In our opinion, *Prosciurus ordosicus* must be assigned to *Ninamys* and is likely a junior synonym of *N. arboraptus*. A direct comparison of the specimens was not possible in the frame of the present study but would be necessary to confirm this synonymy.

By contrast, the single M1/2 identified as *Haplomys arboraptus* by Wang (1987) does not fit the morphology of the type material published by Shevyreva (1971) (see Vianey-Liaud et al. 2013, for a new description and illustration of the type material). This fossil differs from the Mongolian material here studied by its more linguo-labially elongated shape, its absence of hypocone and its antero-posterioly elongated metaconule and by having both ectolophs (paracone and metacone) joining labially and closing central sinus.

*Ninamys kazimierzi* Vianey-Liaud et al., 2013

**Fig. 3:**
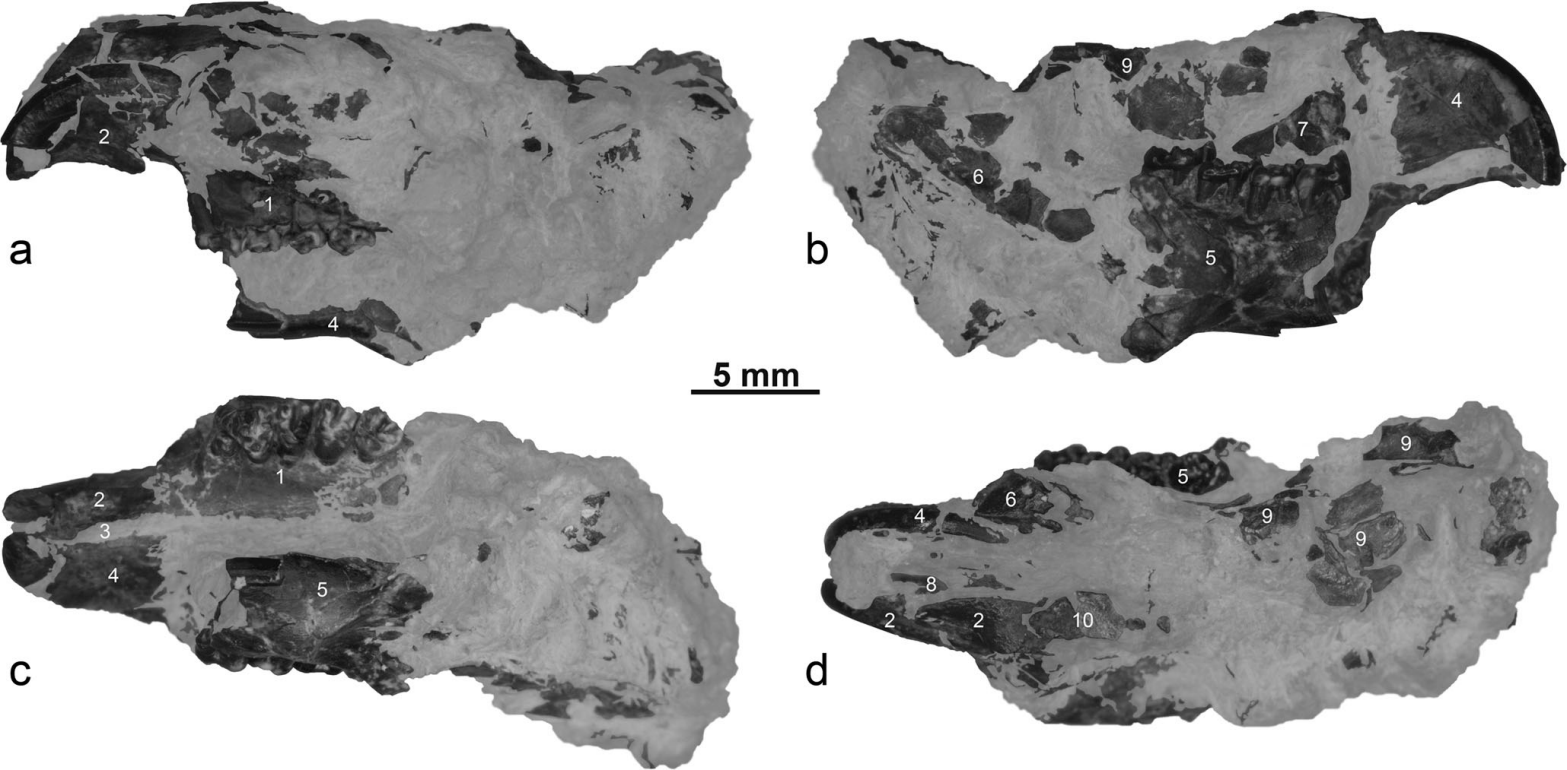
Partial skull of *Ninamys kazimierzi* from the early Oligocene (local biozones B) from the locality Del (DEL-B/8), NHMW 2015/0356/0001 (**a** left view, **b** right view, **c** ventral view, **d** dorsal view): *1*, left maxilla with complete tooth row (P3-M3); *2*, left premaxilla with upper incisor; *3*, incisive foramen; *4*, right premaxilla with upper incisor; *5*, right mandible with complete tooth row (p4-m3); *6*, condyloid process of the mandible; *7*, dorsal part of the right maxilla with fragment of zygomatic arch; *8*, fragment of left nasal bone; *9*, parietal bone; *10*, fragment of frontal bone

**Fig. 4:**
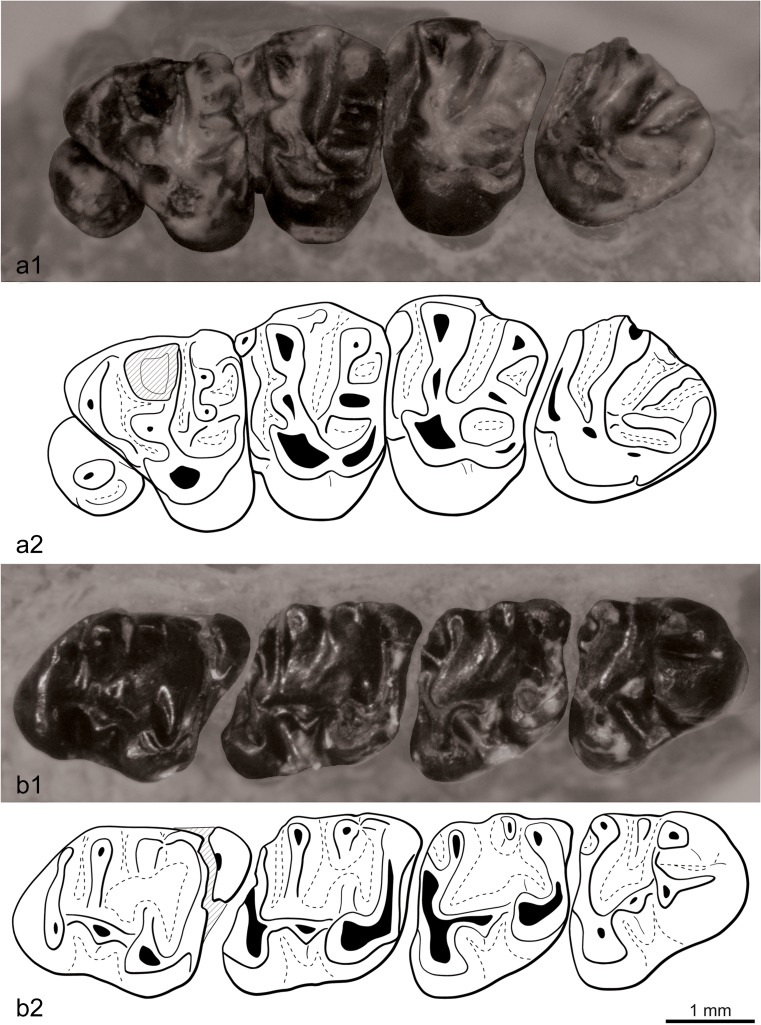
Left upper (P3-M3) and right lower (p4-m3) tooth rows of the skull of *Ninamys kazimierzi* from the early Oligocene (local biozones B) from the locality Del (DEL-B/8), NHMW 2015/0356/0001 (**a** *1* photography of the upper tooth row, **a** *2* sketch drawing of the upper tooth row, **b** *1* photography of the lower tooth row, **b** *2* sketch drawing of the lower tooth row)

**Fig. 5:**
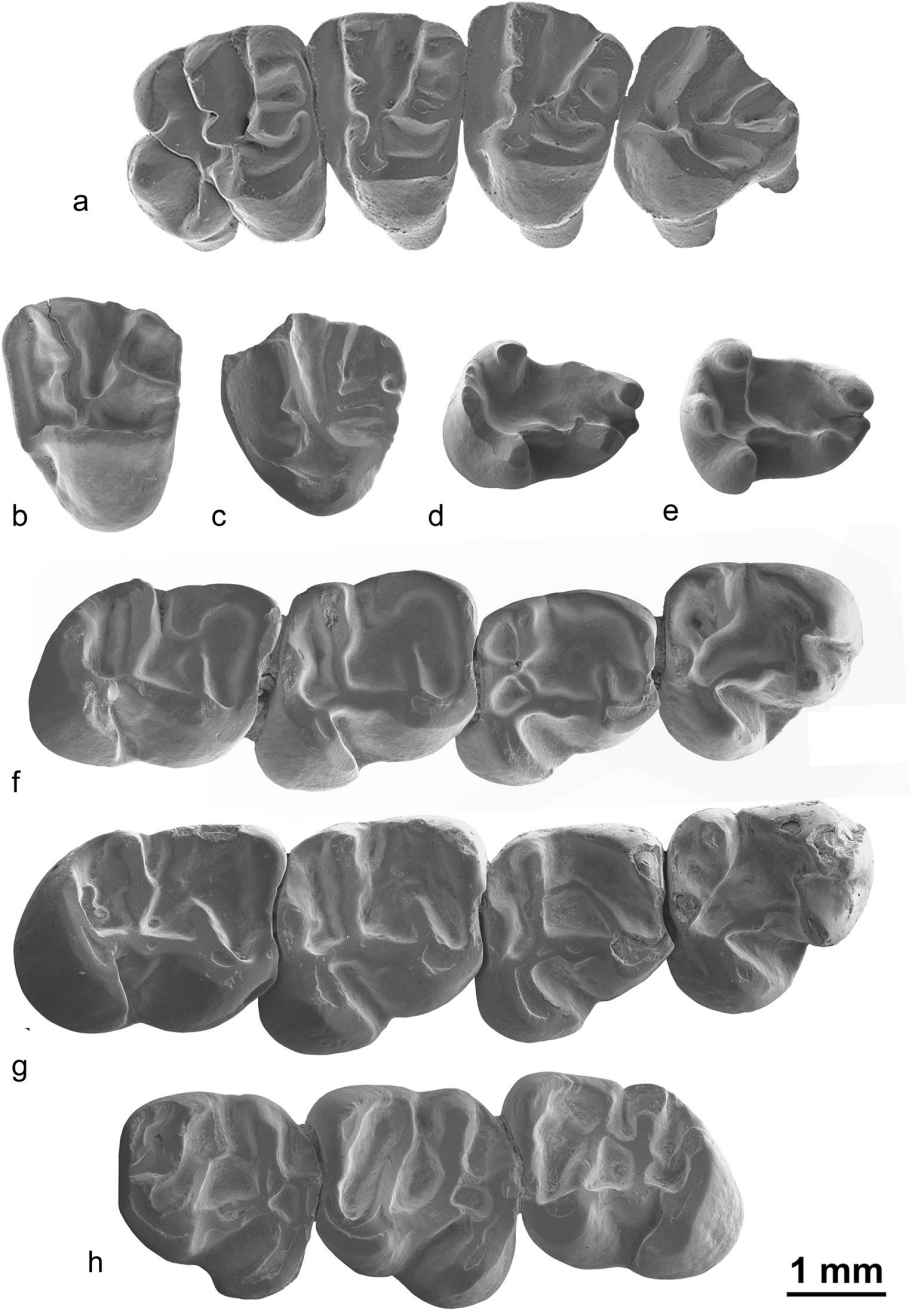
*Nimamys kazimierzi* (**a–g**) and *Prosciurus*? *mongoliensis* (**h**) from the early Oligocene (local biozones A and B) of Taatsiin Gol right (TGR), Tatal Gol (TAT), Del (DEL) and Ikh Argalatyn Nuruu (IKH), occlusal view. *Nimamys kazimierzi*: **a** left maxilla with P3-M2, TGR B/1 (NHMW 2009/0137/0001); **b** left M1/2, TAT-C/3 (NHMW 2015/0348/0001); **c** left M3, TGR-AB/21 (NHMW 2015/0365/0001); **d** right dp4, DEL-B/7 (NHMW 2015/0355/0005); **e** right dp4, DEL-B/7 (NHMW 2015/0355/0004); **f** right mandible with p4-m3, IKH-A/1 + 2 (NHMW 2015/0358/0001); **g** right mandible with p4-m3, TAT-D/1 (NHMW 2015/0349/0001). *Prosciurus*? *mongoliensis*: **h** left mandible with m1-m3, TAT-D/1 (NHMW 2015/0350/0001)

**Fig. 6:**
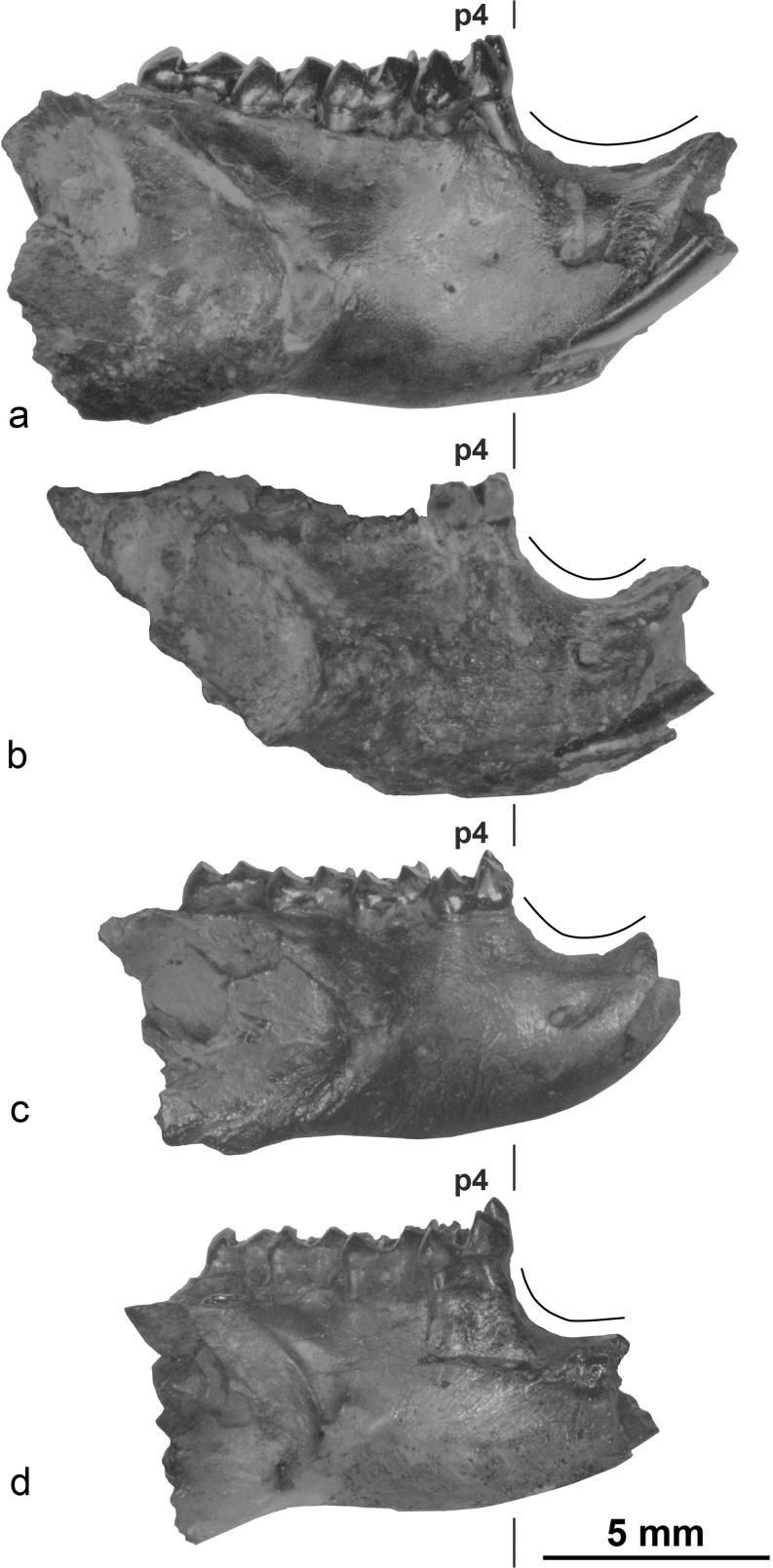
Comparison of mandibles morphology, labial view: **a** *Ninamys kazimierzi*, right mandible with p4-m3, TAT-D/1 (NHMW 2015/0349/0001); **b** *Prosciurus*? sp. nov., left mandible with p4, TAT-D/1 (NHMW 2015/0352/0001) [reversed]; **c** *Promeniscomys* cf. *sinensis*, left mandible with p4-m3, TGR-AB/21 (NHMW 2015/0366/0001) [reversed]; **d** *Proansomys badamae* sp. nov, right mandible with p4-m3, TGR-C/1 (NHMW 2015/0374/0002, holotype)

Figs. 3, 4, 5a–g and 6a

### Synonymy

2010 *Prosciurus* sp.1—Daxner-Höck et al. fig. 4.11, p. 356

**Localities/stratigraphy:** Tatal Gol (layer TAT-D/1, biozone A; layer TAT-C/7, biozone B; early Oligocene); Taatsiin Gol left (layer TGL-A/1, biozone A; TGL-A/11b, biozone B; early Oligocene); Taatsiin Gol right (layer TGR-A/13, biozone A; layers TGR B/1 and TGR-AB/21, biozone B; early Oligocene); Del (layer DEL-B/7 and DEL-B/8, biozone B; early Oligocene); Ikh Argalatyn Nuruu (layer IKH-A/1-2, biozone B; early Oligocene); Unkheltseg (layer UNCH-A/3, biozone B; early Oligocene).

**Material:** 6 isolated upper molars, 9 isolated lower molars, 1 fragment of maxilla, 2 mandibles, 1 partly preserved skull with a complete upper tooth row and a complete lower tooth row; see appendix (ESM) for details.

**Measurements:** Given in Table 3, see appendix (ESM) for details.

**Table 3:** Measurements (in mm) of *Ninamys kazimierzi* from the localities: Tatal Gol (TAT), Taatsiin Gol left (TGL) and Taatsiin Gol right (TGR), local biozone A; Del (DEL), Ikh Argalatyn Nuruu (IKH), Tatal Gol (TAT), Taatsiin Gol left (TGL), Taatsiin Gol right (TGR) and Unkheltseg (UNCH), local biozone B

	L						W/TaW						TrW					
	*N*	Min	Max	Mean	*σ*	CV	*N*	Min	Max	Mean	*σ*	CV	*N*	Min	Max	Mean	*σ*	CV
Biozone A																		
P4	1	–	–	2.15	–	–	1	–	–	2.64	–	–	–	–	–	–	–	–
p4	1	–	–	2.26	–	–	1	–	–	2.24	–	–	1	–	–	1.50	–	–
m1/2	3	2.00	2.40	2.19	–	–	3	2.00	2.30	2.15	–	–	3	1.70	2.00	1.84	–	–
m3	1	-	-	2.51	–	–	1	–	–	1.94	–	–	1	–	–	2.02	–	–
Biozone B																		
P3	2	1.00	1.12	1.06	–	–	2	1.22	1.30	1.26	–	–	–	–	–	–	–	–
P4	2	1.96	2.10	2.03	–	–	2	2.30	2.34	2.32	–	–	–	–	–	–	–	–
M1/2	6	1.56	1.93	1.72	0.14	7.88	5	2.22	2.53	2.38	0.13	5.64	–	–	–	–	–	–
M1	2	1.56	1.71	1.64	–	–	2	2.26	2.45	2.36	–	–	–	–	–	–	–	–
M2	2	1.61	1.81	1.71	–	–	2	2.22	2.45	2.34	–	–	–	–	–	–	–	–
M3	4	1.71	1.98	1.86	0.12	6.54	4	2.00	2.12	2.05	0.05	2.58	–	–	–	–	–	–
dp4	3	1.79	1.95	1.87	–	–	3	1.47	1.58	1.52	–	–	3	1.21	1.23	1.22	–	–
p4	3	2.07	2.14	2.12	–	–	3	1.79	2.12	1.95	–	–	3	1.38	1.56	1.49	–	–
m1/2	7	1.93	2.28	2.10	0.14	6.84	6	1.84	2.12	1.96	0.11	5.85	6	1.53	1.97	1.78	0.17	9.32
m3	2	2.34	2.35	2.35	–	–	2	1.81	1.86	1.84	–	–	2	1.90	2.13	2.02	–	–

*N* number of teeth measured, *Min* minimum, *Max* maximum, *σ* standard deviation, *CV* variation coefficient of Simpson

### Description

**Skull:** One specimen is a partly preserved skull, crushed laterally on both sides, with an associated mandible (NHMW 2015/0356/0001). In dorsal view, the area for both nasal bones is large; part of the parietal bone preserved shows a lateral development indicating a wide basicranum; in dorsal view, the strongly curved rostro-lateral margin of the parietal bone indicates a narrow inter-orbital area. In ventral view, the incisive foramina is short and seems to end caudally much before the maxillary bone. A fragment of the zygomatic arch is preserved; it is thin with a rounded section.

**P3:** The tooth is rounded, composed of a single cusp with a long cingulum starting posteriorly and forming a loop on the lingual edge; the cingulum delimits a narrow lingual sinus.

**P4**: The anterior lobe is relatively short and narrow. The anterocone, protocone, paracone, metacone, metaconule and paraconule are all well-developed, but the hypocone is weakly developed. A very short anterior arm of the protocone is present. A bulge of the lingual part of the anteroloph, between the anterocone and the protocone, suggests the presence of a poorly developed anterostyle. The thick labial anteroloph connects the anterocone to the base of the paracone, possibly including a parastyle, but it is not observable due to the degree of wear of the tooth. The posteroloph is complete between the hypocone and the metacone, low and weakly developed. The lingual metaloph connects on the lingual protoloph between the paraconule and the protocone. The labial border is broken, so the ectoloph and mesostyle are not observable. The roots are not preserved.

**M1/2**: All cusps are well-developed. A very short anterior arm of the protocone is present. The anterostyle is either poorly developed or absent, whereas the parastyle is always present but weakly developed. The mesostyle is well-developed and the mesoloph is absent. The mesial part of the ectoloph (between the paracone and the mesostyle) is always present and weakly developed. The posterior part of the ectoloph (between the mesostyle and the metacone) is either absent or weakly developed, but it is never more developed that the anterior part. The posteroloph is complete and well-developed; the metaconule is antero-posterioly elongated and connects to the posteroloph. The lingual metaloph connects either on the lingual protoloph between the paraconule and the protocone or directly on the paraconule. The tooth has three roots.

**M3:** The anterostyle is well-developed and large, although not as large as the protocone, paracone and metacone. The anteroloph is usually complete, but in one case (NHMW 2015/0355/0003), it is interrupted lingually and does not connect to the anterostyle. The paraconule is well-developed; in one case (NHMW 2015/0355/0003), it is anteriorly extended and almost reaches the anteroloph. Both lingual metaloph and lingual protoloph merge to form a single loph reaching the protocone. The hypocone can be either absent or well-developed. The metaconule is antero-posteriorly elongated and connects to the posteroloph. The central sinus is closed labially by the ectoloph. The ectoloph is either equally developed or more developed on the paracone side (like in M1/2); there is no mesostyle. The tooth has three roots.

**Mandible:** The mandible is relatively robust (compared to other aplodontid taxa found in the Valley of Lakes; Fig. 6), with a shallow and weakly curved diastema. The coronoid process is not preserved, but the base is placed close to the posterior part of the m3. Both the dorsal and the ventral masseter crests are weak. The dorsal masseter crest joins the ventral one at a point below the middle of m2. The ventral masseter crest extends even further, extending ventral to the m1. The ventral masseter also extends ventrally below the body of the mandible. The position of the mental foramen is relatively dorsal, such as it is visible in the occlusal view. In the diastema, the mental foramen is located posteriorly, more or less near the anterior root of the m1.

**dp4:** Both the metalophid I and II are present but incomplete and do not always reach the metaconid. A long metastylid crest is attached to the metaconid, but the mesostylid and entoconid ridge are absent so the talonid basin is open lingually. The mesoconid is well-developed but smaller than the other cuspids; in lateral view, it is also noticeably lower. The entolophid can be either complete or absent, but the entoconulid is not visible. The hypoconulid is usually well-developed and as high as the other cuspids in lateral view, although it is not visible on one tooth (NHMW 2015/0363/0001). When the entolophid is present, there is no lophid connecting the hypoconulid to the entolophid so the posterior sinusid is not divided. The tooth has two roots.

**p4:** In lateral view, the protoconid and metaconid are higher than the other cuspids. The metalophid II is well-developed and links the metaconid to the protoconid, whereas the metalophid I is either absent or incomplete. The metastylid crest is always well-developed, but the mesostylid and mesolophid are absent. In some teeth (2/3), the metastylid crest does not reach the entoconid so the talonid basin is open lingually. The entolophid is either absent or forms a loop and connects directly the entoconid to the hypoconulid, but it never connects to the ectolophid, and the entoconulid is always absent. In one tooth (NHMW 2015/0349/0001), a small anterior spur starts from the entolophid but ends freely in the talonid basin. The hypoconid is oblique and elongated anterior-labially. One tooth shows a well-developed labial cingulum closing the sinusid, whereas it remains open in other teeth. The tooth has two roots.

**m1/2:** In lateral view, the protoconid and metaconid are higher than the other cuspids. The anteroconulid is weakly developed and appears to be merging with the protoconid, depending on how worn the tooth is. The metastylid crest is always present, from weakly to well-developed. Likewise, the mesostylid is always present, but either weakly or well-developed. The mesolophid shows a remarkable variability, it is either absent or short and ends freely in the talonid basin or long and connects to the entoconulid. The metalophid II is well-developed and long; it usually ends free in the talonid basin on the m2s. It is more variable on the m1s: it can either end free in the talonid basin or be connected to the mesolophid. The hypoconulid is always well-developed, whereas the entoconulid can be either well-developed or absent; the entoconulid crest connecting them can be either complete, incomplete or absent. The mesoconid is either weak or well-developed; the ectomesolophid is either weakly developed or absent. The entolophid connects to the ectolophid, posteriorly to the mesoconid, in m2s but not always in m1s. The hypoconid is oblique and anterior-labially elongated. The teeth usually have two roots, but one specimen (NHMW 2015/0353/0001) shows two pulp cavities and a posterior sinus on the anterior root.

**m3:** Slightly longer than the m2. In lateral view, the protoconid and metaconid are higher than the other cuspids. The anteroconulid is weakly developed or absent. Sometimes the anteroconulid presents a short anteroloph forming a loop on the lingual edge. The metalophid I is usually complete but almost interrupted lingually to the anteroconulid in one specimen (NHMW 2015/0349/0001). The metaconid is slightly smaller than the three other main cuspids; it is prolonged posteriorly by a well-developed metastylid crest reaching the mesostylid. The mesostylid is also well-developed with a mesolophid which can be short or long. The mesolophid either ends free on the talonid basin (2/3) or bends posteriorly to connect with the entoconulid or the entolophid (1/3). The entoconulid can be either well-developed or absent. The hypoconulid is absent (or too weakly developed to be visible); the entoconulid crest is either absent or starts from lingual posterolophid and ends freely in the posterior sinusid. The entolophid connects to the mesoconid or posteriorly to the mesoconid; the ectomesolophid is weakly developed or absent. The hypoconid is oblique and anterior-labially elongated. The tooth has two roots.**Comparisons and discussion:** The partly preserved skull shows some features characteristic of the family Aplodontidae found also in extant relatives of the family, like *Aplodontia rufa* (Rafinesque 1817) such as the wide basicranum, the narrow inter-orbital region, the wide nasal area, the short incisive foramen and the thin rounded section of the zygomatic arch (protogomorphous condition). The morphology of the cheek teeth found in Mongolia generally fits the diagnosis of *Ninamys* as given by Vianey-Liaud et al. (2013). Furthermore, the discovery of a partial skull with associated lower and upper tooth rows allows us to confirm the correspondence of lower and upper dental characters for this species. But, as for *N. arboraptus*, the specimens described above show more morphological variability than expected, especially for the ectoloph of the upper molars of *Ninamys*.

At the species level, according to Vianey-Liaud et al. (2013), *N. kazimierzi* differs from *N. arboraptus* by the higher crown of the cheek teeth, the absence of the hypocone on upper molars, the stronger protocone and wear facets diving more steeply. Our observations confirm that the height of the crown and the wear facets allow a clear differentiation from *N. arboraptus*, whereas the development of the protocone and hypocone shows more variability and does not seem to be reliable characters to differentiate the two species for the material of Central Mongolia. However, a few additional characters seem to help in differentiating the two species in our material, such as the size (*N. kazimierzi* is slightly larger than *N. arboraptus*), the length of the m3 (slightly longer that the m2 in *N. kazimierzi* whereas m3 and m2 are about the same size in *N. arboraptus*) and the development of the anterior lobe on the P4 (proportionally shorter and narrower in *N. kazimierzi*).

Genus *Prosciurus* Matthew, 1903

*Prosciurus*? *mongoliensis* Wang and Dashzeveg, 2005

Fig. 5h

**Localities/stratigraphy:** Tatal Gol (layer TAT-D/1, biozone A; early Oligocene).

**Material and measurements:** One fragment of left mandible with m1-m3 (NHMW 2015/0350/0001); m1 (L = 1.95; TrW = 1.57; TaW = 1.97 mm); m2 (L = 2.30; TrW = 1.84; TaW = 2.16 mm); m3 (L = 2.47; TrW = 1.86; TaW = 1.83 mm).

### Description

**Mandible:** The specimen is broken rostrally to the m1 and caudally to the m3, so most of the morphology is not observable. The coronoid process is not preserved, but its base starts close to the middle part of the m3.

**m1:** The protoconid is anteriorly elongated and the anteroconulid is not visible. The metalophid I is not connected to the metaconid but it is curved and connects posteriorly to the metaconid, on the metastylid crest. Due to the shape of the metalophid I, an anterior sinusid is formed between the metalophid I and the metaconid and opens anteriorly. The metalophid II is long and almost parallel to the entolophid; it is connected to the posterior extremity of the metastylid crest (possibly including the mesostyle). A thin, low crest connects the metalophid II and the entoconid, closing the talonid basin. The hypoconid is labially elongated. The mesoconid is well-developed but small. The entolophid is connected on the ectolophid, posterior to the mesoconid. The entoconulid and the hypoconulid are all well-developed and linked together by the entoconulid crest. The posterolophid is short and does not reach the entoconid, leaving the posterior sinusid lingually open. The tooth has two roots.

**m2:** The morphology is similar to the m1; but the metalophid I connects directly to the anterior part of the protoconid and the metaconid. The posterolophid is longer than in m1 and reaches the entoconid. Also, the entolophid is joined to the mesoconid. Apart from this, the anterior part of the tooth is wider. The protoconid is anteriorly elongated and the anteroconulid is not visible. The metalophid II is long and almost parallel to the entolophid; it connects to the posterior extremity of the metastylid crest (possibly including the mesostyle). A thin, low crest connects the metalophid II to the entoconid, closing the talonid basin. The hypoconid is labially elongated. The mesoconid is well-developed but small. The entoconulid and the hypoconulid are well-developed and connected by the entoconulid crest. The tooth has two roots.

**m3:** The tooth is longer than the m2. The metalophid I connects directly the anterior part of the protoconid to the metaconid. The protoconid is not anteriorly elongated as in the m1 and m2, and the anteroconulid is absent. The metalophid II is long and longitudinally oriented; it connects to the posterior extremity of the metastylid crest (it may include the mesostyle). A crest, more developed than in m1 and m2, connects the metalophid II to the entoconid, closing the talonid basin. The hypoconid is labially elongated. The mesoconid is well-developed but small. The entolophid is connected to the mesoconid. The entoconulid and the hypoconulid are well-developed, but the entoconulid crest linking them is incomplete. The posterolophid reaches the entoconid. The roots are not clearly visible.

**Comparisons and discussion:** The size and morphology of this specimen match the diagnosis of *Prosciurus mongoliensis* made by Wang and Dashzeveg (2005). In this diagnosis, Wang and Dashzeveg (2005) did not compare *Prosciurus mongoliensis* with *N. arboraptus* despite of their close size. However, *Prosciurus mongoliensis* is slightly larger than *N. arboraptus*. Apart from that, the morphology allows a clear distinction between these species. *Prosciurus mongoliensis* differs from *N. kazimierzi* by its weak mesoconid, its absent ectomesolophid, its well-developed entoconulid and hypoconulid which are linked by an entoconulid crest (although it can be incomplete), and by its long metalophid II, longitudinal to the entolophid, that reaches the posterior edge of the metastylid crest.

Recently, Vianey-Liaud et al. (2013) reviewed *Prosciurus*, emending its diagnosis. They also questioned the assignment of the Asian representatives of this genus although they did not propose an alternative assignment. Among the diagnostic features included in the emended diagnosis of *Prosciurus* (Vianey-Liaud et al. 2013, p. 85), several are different from the material here studied such as the following: the m3 as long as m2, the short metalophulid II, the mesostylid separated from metaconid and the entoconid with distinct entolophid bending posteriorly towards posterolophid. Also, they indicated in the emended diagnosis of *Prosciurus* that the upper teeth lack the ectoloph, have a small hypocone, two metaconules, the lingual part of the metaloph should be sometimes absent, or complete but connected directly to the protocone. None of the upper molars of Central Mongolia have this morphology, and this could lead us to preclude the assignment of the Mongolian material to the genus *Prosciurus*. However, the upper teeth of *Prosciurus mongoliensis* are unknown so far, so we can neither fully confirm nor reject the assignment of *Prosciurus mongoliensis* to *Prosciurus*. Consequently, especially with the upper dentition missing, the question of the generic attribution of this species remains open.

*Prosciurus*? sp. nov. Wang and Dashzeveg, 2005

Fig. 6b

### Synonym

2005 *Prosciurus* cf. *mongoliensis*—Wang and Dashzeveg fig. 4, p. 96.

**Localities/stratigraphy:** Tatal Gol (layer TAT-D/1, biozone A; early Oligocene).

**Material and measurements:** Fragment of left mandible with p4 (NHMW 2015/0352/0001; L = 2.10, TrW = 1.49, TaW = 2.19 mm).

### Description

**Mandible:** The mandible is slender, with a deep and strongly curved diastema. The coronoid process is not preserved, but its base shows an origin lateral to the posterior part of m3. Both the dorsal and the ventral masseter crests are weak. The dorsal masseter crest joins the ventral one at a point ventral to the proximal part of m2. The ventral masseter crest extends even further, to a point ventral to the m1. The position of the mental foramen is shifted so far dorsally that it is visible in the occlusal view. It is located close to the middle of the diastema where the diastema is at the lowest height.

**p4**: In lateral view, the metaconid is slightly higher than the other cuspids. The metalophid II is short but well-developed and links the metaconid to the protoconid. The anterior part of the tooth is damaged, and the metalophid I is not clearly observable but seems to be present. The metastylid crest and the mesostylid are well-developed but the mesolophid is absent. The metastylid crest does not reach the entoconid so the talonid basin is open lingually. The entolophid forms a loop and connects directly the entoconid to the hypoconulid; the entoconulid is absent. The mesoconid is large but there is no ectomesolophid. The hypoconid is oblique and elongated anterior-labially. The roots are not observable.

**Comparisons and discussion:** The premolar from TAT-D/1 has similar size as *N. kazimierzi* and also, as the specimen of *Prosciurus*? *mongoliensis* presented above (slightly larger than *Prosciurus* cf. *mongoliensis* measured by Wang and Dashzeveg 2005). However, the mandible form TAT-D/1 differs from those of *N. kazimierzi*, by being more slender and by presenting a deep and strongly curved diastema (Fig. 6). The mandible from TAT-D/1 differs from *Prosciurus*? *mongoliensis* because it displays a shorter and more strongly curved diastema, the lower incisor has a triangular cross-section, and the dorsal masseter insertion is also comparatively weaker. The p4 from TAT-D/1 shows a larger mesoconid than *Prosciurus*? *mongoliensis* and a shorter metalophid II. By contrast, these features are present in *Prosciurus* cf. *mongoliensis* described by Wang and Dashzeveg (2005). Although the p4 from Mongolia does not have ectomesolophid and it is slightly larger, we tentatively assigned it to the same taxon, *Prosciurus* cf. *mongoliensis*, described by Wang and Dashzeveg (2005). As previously stated by Wang and Dashzeveg (2005), the fossil record of the Valley of Lakes confirms that two taxa, both tentatively referred to *Prosciurus*, coexist in the same layer (TAT-D/1). Considering the morphological differences between the present specimen and *Prosciurus*? *mongoliensis* (especially on the mandible), we can now confirm that it represents a new species for which affinity with *Prosciurus*? *mongoliensis* is not clearly established. However, the material is so far insufficient to propose a reliable diagnosis. Furthermore, as for *Prosciurus*? *mongoliensis*, the question of the generic attribution remains unsolved. We consequently leave the nomenclature open, *Prosciurus*? sp. nov.

Subfamily Meniscomyinae Rensberger, 1981

Genus *Promeniscomys* Wang, 1987

**Type species:** *Promeniscomys sinensis* (Wang, 1987)

**Emended diagnosis** (modified after Wang 1987 and Vianey-Liaud et al. 2013): Small Aplodontidae, close to *Prosciurus* in size. Cheek teeth brachydont and bunodont. Upper cheek teeth with an always distinct parastyle. Anterostyle either absent, weak or distinct. Both anterior and central sinuses closed labially. Paraconule and metaconule antero-posteriorly elongated and the lingual metaloph connects on the paraconule. Both labial and lingual protolophs joined to the posterior part of the paraconule. Ectoloph well-developed and complete, but the paracone part of the ectoloph thicker. Mesostyle antero-posteriorly compressed and lingually protruded. External cingulum bordering the paracone and the metacone lingually; anterior arm of the protocone weak or absent and hypocone absent. Lower cheek teeth antero-posteriorly elongated with a weak difference between the trigonid and talonid widths and a slight labial inflection. Second molar is the longest. Mesoconid weakly developed on the m1-m3, labio-lingually compressed on the p4. Hypoconid elongated, pointing antero-labially, prolonged by a long ridge pointed anteriorly towards the protoconid and almost closing the labial sinusoid. Entoconulid absent. The metalophid II is long, oriented towards the entoconid and not reaching the metaconid, but connected to the entolophid by a small accessory crest on the m2-m3. Metastylid crest is short and low, not reaching the entoconid so the trigonid basin remains open lingually.

*Promeniscomys* cf. *sinensis*

Figs. 6c and 7

**Fig. 7:**
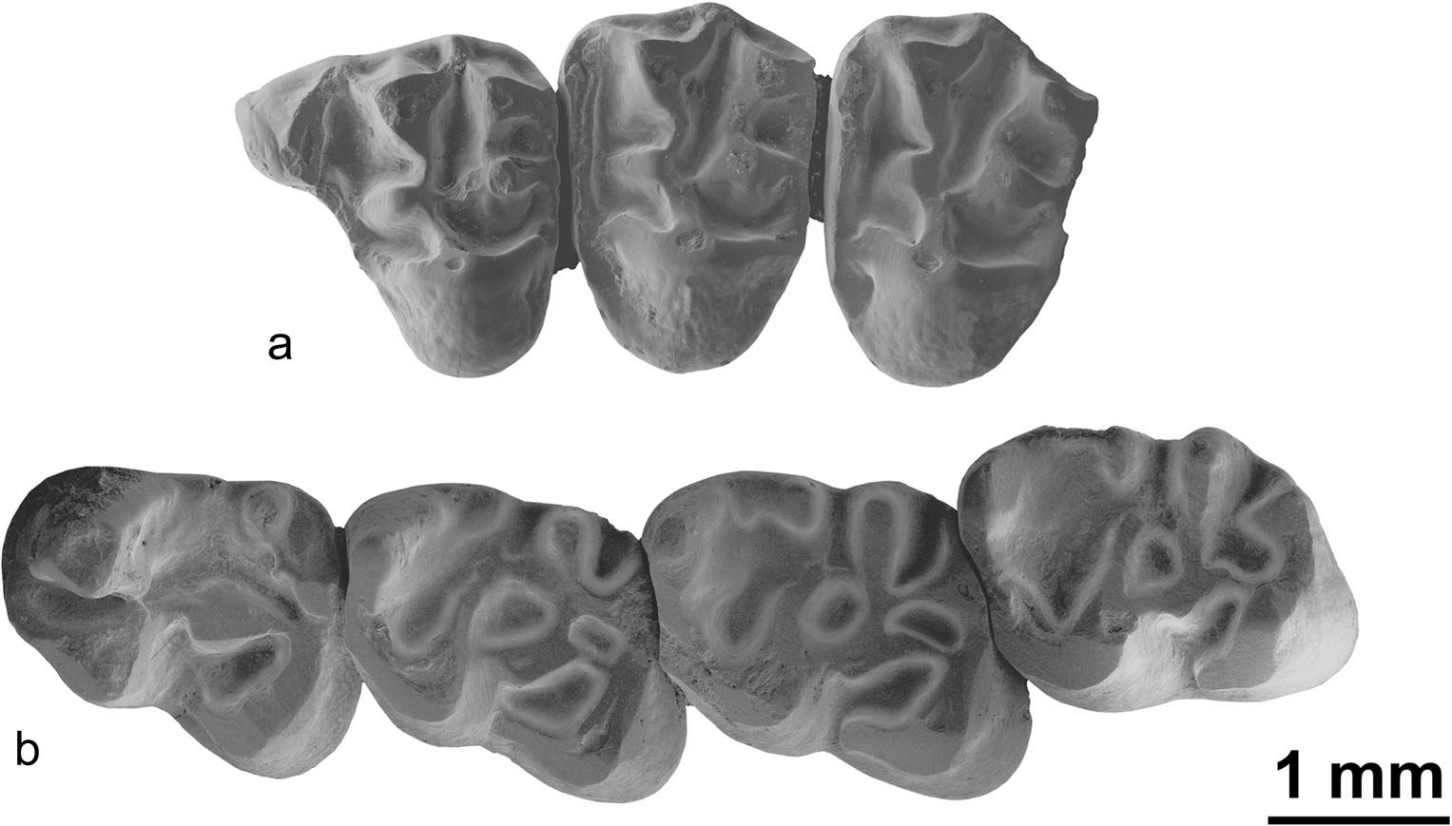
*Promeniscomys* cf. *sinensis* from the early Oligocene localities Taatsiin Gol right (*TGR*) and Tatal Gol (*TAT*), local biozones A and B, occlusal view: **a** fragment of left maxilla with P4-M2, TAT-D/1 (NHMW 2015/0351/0001); **b** left mandible with p4-m3, TGR-AB/21 (NHMW 2015/0366/0001)

**Localities/stratigraphy:** Tatal Gol (layer TAT-D/1, biozone A; early Oligocene); Ikh Argalatyn Nuruu (layer IKH-A/1-2, biozone A; early Oligocene); Taatsiin Gol right (layer TGR-AB/21, biozone B; early Oligocene).

**Material**: Fragment of left maxilla with P4-M2 (NHMW 2015/0351/0001); fragment of right P4 (NHMW 2015/0359/0001); right M1/2 (NHMW 2015/0361/0001); fragment of left M1/2 (NHMW 2015/0368/0001); left mandible with p4-m3 (NHMW 2015/0366/0001).

**Measurements:** Given in Table 4, see appendix (ESM) for details.

**Table 4:** Measurements (in mm) of *Promeniscomys* cf. *sinensis* from the localities: Tatal Gol (TAT), local biozone A; Ikh Argalatyn Nuruu (IKH), Tatal Gol (TAT) and Taatsiin Gol right (TGR), local biozone B

	L				W/TaW				TrW			
	*N*	Min	Max	Mean	*N*	Min	Max	Mean	*N*	Min	Max	Mean
Biozone A												
P4	1	–	–	1.77	1	–	–	1.95	–	–	–	–
M1/2	2	1.44	1.57	1.51	2	2.10	2.20	2.15	–	–	–	–
M1	1	–	–	1.44	1	–	–	2.10	–	–	–	–
M2	1	–	–	1.57	1	–	–	2.20	–	–	–	–
Biozone B												
M1/2	1	–	–	1.52	1	–	–	2.05	–	–	–	–
p4	1	–	–	2.07	1	–	–	1.67	1	–	–	1.32
m1/2	2	2.14	2.26	2.20	2	1.60	1.71	1.66	2	1.46	1.55	1.51
m3	1	–	–	2.20	1	–	–	1.51	1	–	–	1.58

*N* number of teeth measured, *Min* minimum, *Max* maximum

### Description

**P4:** The anterocone is as large as the paracone, with a distinct parastyle, whereas the anterostyle seems absent. The anterior sinus is closed labially by a crest that connects the parastyle and the paracone. The paraconule and metaconule are antero-posteriorly elongated; the metaconule is prolonged by a lingual metaloph that connects on the paraconule. The protoloph is straight and is joined to the posterior part of the paraconule, which is labio-lingually compressed and reaches the anteroloph. The protocone is prolonged anteriorly by the anteroloph, the anterior arm of the protocone is absent. Both paracone and metacone parts of the ectoloph are well-developed, but the paracone part is thicker. The ectoloph is as thick as the paracone and both present a characteristic oblique wear surface oriented posteriorly. Both parts of the ectoloph merge on the mesostyle which is protruding lingually, antero-posteriorly compressed, and closes the central sinus. An external cingulum surrounds the paracone and the metacone lingually. The hypocone is absent. The tooth has three roots.

**M1/2:** The M1 and M2 are very similar, the M2 is slightly larger. The parastyle is well-developed, linguo-labially elongated, forming part of the anteroloph; the anterostyle is either absent or weakly developed, and the anterior arm of the protocone is absent on the M1 and very weak on the M2. The paraconule and metaconule are antero-posteriorly elongated; the lingual metaloph starts from the metaconule and is connected to the paraconule. The protoloph is straight but, unlike P4, the paraconule is crescent-like, both labial and lingual protolophs join to the posterior part of the paraconule. As on the P4, the parastyle connects to the paracone so the anterior sinus is closed labially. Both paracone and metacone parts of the ectoloph are well-developed, the paracone part is thicker. The ectoloph departs from the paracone. Both have a characteristic oblique wear surface oriented posteriorly. Both parts of the ectoloph are joined to the labial part of the mesostyle which is protruding lingually, antero-posteriorly compressed. The central sinus is closed by the mesostyle and the ectoloph. A cingulum surrounds the paracone and the metacone lingually. Both M1 and M2 display a short lingual anteroloph delimitating a narrow lingual sinus antero-lingually to the protocone. The hypocone is absent. The tooth has three roots.

**Mandible:** The mandible is slender, with a shallow and weakly curved diastema. The coronoid process is not preserved, but its base is situated lateral to the posterior part of m3. Both dorsal and ventral masseter crests are well marked. The dorsal masseter crest joins the ventral one below the posterior part of m2. The ventral masseter crest is stronger than the dorsal one and extends slightly further anteriorly, below the proximal part of the m2. The position of the mental foramen is low, barely visible in the occlusal view. The mental foramen is located close to the middle part of the diastema, at its lowest part.

**p4:** The tooth is elongated, with the outline almost rectangular and the talonid is only slightly wider than the trigonid. In lateral view, both the protoconid and metaconid are the highest cusps. A crest starts anteriorly from the protoconid and bends towards the metaconid, which could be interpreted as an incomplete metalophid I that does not reach the metaconid. The anteroconulid is absent. The metalophid I is short and complete.

The metastylid crest is weakly developed and low so the talonid basin is widely open lingually. The mesoconid is well-developed and labio-lingually compressed. The hypoconid is elongated, antero-labially directed; it is prolonged by a long ridge directed anteriorly towards the protoconid and almost closing the labial sinusid. This hypoconid ridge shows strong oblique wear surface oriented anteriorly. The entolophid connects directly the entoconid to the hypoconulid, and the entoconulid is absent. The posterolophid is short and thin, and it connects to the base of the entoconid enclosing a small lingual posterior sinusid. The tooth has two roots.

**m1/2:** The m1 is slightly longer than the p4. The m2 is the longest molar of the tooth row. Both m1 and m2 are elongated with the talonid only slightly wider than the trigonid and a slight labial inflection. The metaconid is located much more anteriorly compared to the protoconid. The metalophid I is complete. An anteroconulid is not present between the protoconid and the metaconid. The metalophid II is long but does not reach the metaconid; it is postero-lingually directed towards the entoconid. The metalophid II on the m1 ends free in the trigonid basin, whereas on the m2, it is connected to the entolophid by a small accessory crest. The metastylid crest is short and low, and it does not reach the entoconid so the trigonid basin remains open lingually. The m2 shows a big mesostylid at the edge of the metastylid crest, but this cusp is not present on the m1. The hypoconid is elongated and antero-labially directed; it is prolongated by a long ridge pointed anteriorly towards the protoconid and almost closing the sinusid. As in p4, the hypoconid ridge is marked by a strong oblique wear surface oriented anteriorly. The entolophid is divided and links the entoconid to both the hypoconulid and the ectolophid. The mesoconid is weakly developed. The entoconulid is absent. The hypoconulid is well-developed and prolonged by a short, thin posterolophid reaching the base of the entoconid. The tooth has two roots.

**m3:** The m3 is slightly shorter than the m2 and slightly longer than the m1. The talonid width is almost equal to the trigonid width, so the outline of the tooth is subrectangular. Otherwise, the general morphology of the tooth is very similar to the m2. It only differs by the presence of a very small anteroconulid, an incomplete crest between the hypoconulid and the entolophid and a narrowing of the ectolophid between the entolophid and the hypoconid. The tooth has two roots.

**Comparisons and discussion:** The above-described specimens present several morphologic features typical of the subfamily Meniscomyinae such as the following: on upper cheek teeth: the paraconule reaching the anteroloph; the continuous ectoloph with a mesostyle antero-posteriorly compressed and connected to the labial part of the meseostyle which is protruding lingually; single metaconule, and absent hypocone. On lower cheek teeth: the labial inflection; the long ridge prolonging the hypoconid labially; the strong mesostylid, and the long metalophid II postero-lingually directed and connected to the entolophid. The upper and lower cheek teeth are of similar sizes. Besides, the hypoconid ridge on lower molars and the ectoloph on the upper molar both display strong oblique wears surfaces (respectively oriented anteriorly and posteriorly), fitting each other in the occlusion of teeth. Therefore, we assign them to the same species.

Regarding the morphology, the upper molars here studied have a large anterocone on P4, a small posterior labial sinus, a mesostyle antero-posteriorly compressed and an external lingual cingulum surrounding the paracone and the metacone. These features are diagnostic of the genus *Promeniscomys* as described by Wang (1987) that belongs to the subfamily Meniscomyinae. Our specimen differs from the original diagnosis of *Promeniscomys* in having the paraconule reaching the anteroloph and missing the well-developed anterostyle. However, we still refer the specimen to *Promeniscomys* and rather attribute these few differences to the morphological variability at generic level. We have emended the diagnosis (see above) in order to include all the intraspecific variability of the genus.

*Promeniscomys* is so far a monospecific genus. The species *Promeniscomys sinensis* is poorly known. It was first described by Wang (1987) based on a single fragmentary maxilla bearing P3-M1 from the Wulanbulage Formation in Inner Mongolia (China). Vianey-Liaud et al. (2013) proposed an emended diagnosis of the species based on a single additional P4 discovered in Ulantatal (Vianey-Liaud et al. 2013, p. 101). The new specimens discovered in Central Mongolia provide important information about previously unknown elements like the lower jaw with p4-m3. The morphology of our upper cheek teeth is very similar to that of the type material. However, they differ slightly in having a straight protoloph, paraconule reaching the anteroloph on the P4 and the lingual metaloph connecting on the paraconule. These differences may be either part of the intraspecific variability of *Promeniscomys sinensis* or a new species. Unfortunately, the Mongolian material is so far insufficient to state it clearly. Therefore, we tentatively refer our specimens to the type species *Promeniscomys* cf. *sinensis*.

It is noteworthy that the morphology of the lower cheek teeth confirms that *Promeniscomys* is closely related to the American genus *Meniscomys*, as already stated by Wang (1987) based on upper cheek teeth.

Subfamily Ansomyinae Qiu, 1987

Ansomyinae indet.

Fig. 8a

**Fig. 8:**
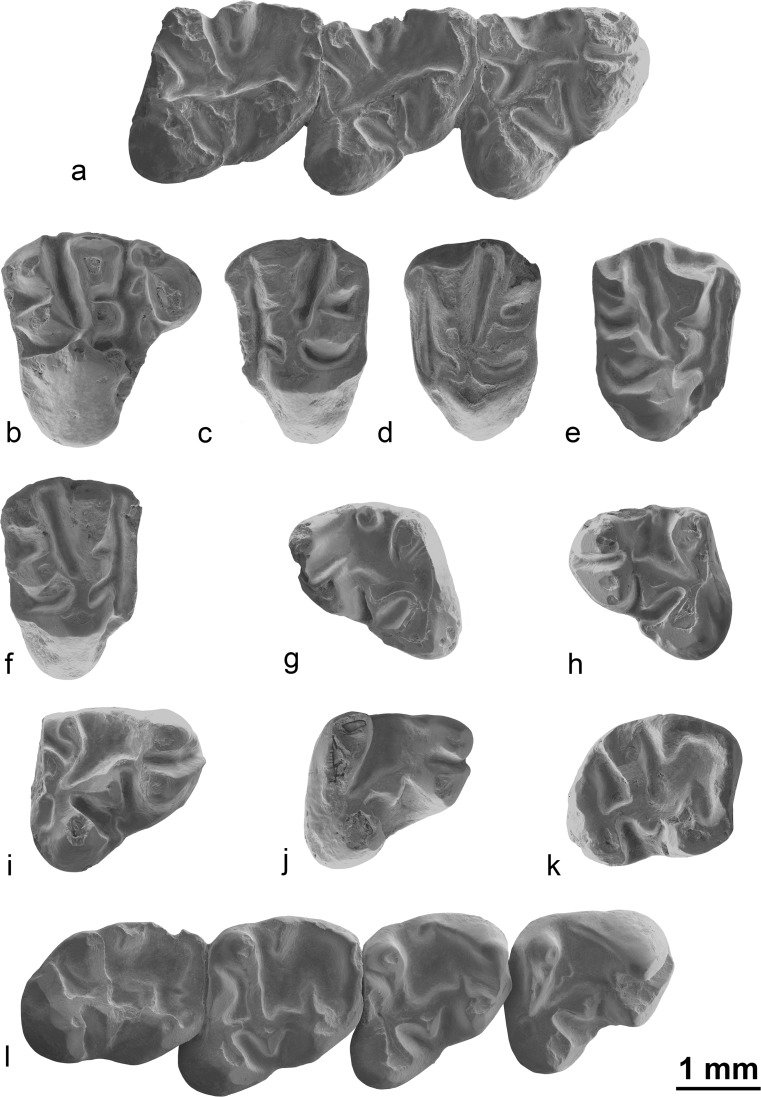
Ansomyinae indet. (**a**) and *Proansomys badamae* sp. nov. (**b–l**) from the late Oligocene (local biozones C and C1) of the locality Taatsiin Gol right (TGR), occlusal view. Ansomyinae indet.: **a** right mandible with p4-m2, TGR-C/1 (NHMW 2015/0373/0001). *Proansomys badamae* sp. nov.: **b** right P4, TGR-C/7 (NHMW 2015/0381/0001); **c** left M1/2, TGR-C/7 (NHMW 2015/0381/0003); **d** left M1/2, TGR-C/7 (NHMW 2015/0381/0004); **e** right M1/2, TGRC/2 (NHMW 2015/0376/0001); **f** right M1/2, TGR-C/7 (NHMW 2015/0381/0005); **g** left m1, TGR-C/2 (NHMW 2015/0376/0005); **h** left p4, TGRC/2 (NHMW 2015/0376/0004); **i** right p4, TGR-C/1 (NHMW 2015/0374/0001); **j** right p4, TGR-C/7 (NHMW 2015/0381/0007); **k** right m3, TGR-C/7 (NHMW 2015/0381/0008); **l** right mandible with p4-m3, TGR-C/1 (NHMW 2015/0374/0002, holotype)

**Localities/stratigraphy:** Taatsiin Gol right (layers TGR-C/1 and TGR-C/2, biozone C, early late Oligocene).

**Material and measurements**: Right mandible with p4-m2 (NHMW 2015/0373/0001; p4, L = 2.26, TrW = 1.52, TaW = 2.14 mm; m1, L = 2.35, TrW = 1.87, TaW = 1.97 mm; m2, L = 2.19, TrW = 1.58, TaW = 1.88 mm); fragment of left mandible with m2-m3, strongly worn (NHMW 2015/0375/0001; m2, L = 2.36, TrW = 1.84, TaW = 2.05 mm; m3, L = 2.40, TrW = 1.77, TaW = 1.67 mm).

### Description

**Mandible:** The mandibles are slender, with a shallow and slightly curved diastema. The coronoid processes are not preserved, but their base is situated lateral to the posterior part of m3. Both dorsal and ventral masseter crests are weakly marked. The dorsal masseter crest joins the ventral one below the middle part of m2. The dorsal masseter crest is stronger than the ventral one, but the ventral masseter extends slightly further anteriorly, below the posterior part of the m1. The position of the mental foramen is low but still visible in the occlusal view. The mental foramen is located close to the middle part of the diastema, at its lowest part.

**p4:** Both protoconid and metaconid are close, delimitating a very narrow valley between them. The metalophid I is interrupted and does not reach the metaconid, whereas the metalophid II is short but complete. The anteroconulid is absent. The metastylid crest is long but does not close the talonid basin. Both mesoconid and ectomesolophid are strong; the ectomesolophid has a triangular shape. A strong oblique entolophid connects directly the entoconid to the ectolophid, in a point close to the mesoconid. The entoconulid is absent, whereas the hypoconulid is strongly developed. A short labial posterolophid is present; it forms a loop starting from the hypoconulid and connecting to the hypoconid in its lingual part. There is no connection between the hypoconulid and the entoconid so the posterior sinusid is open postero-lingually. The roots are not visible.

**m1/2:** The first and second molars only differ by their size (the m1 being noticeably smaller) and the relative position of the protoconid and metaconid (both cusps being close to each other on the m1). Otherwise, the protoconid and metaconid are linked by a short and thick metalophid I, whereas the metalophid II is absent. The anteroconulid is absent or not distinguishable from the metalophid I. The mesostylid is strong and the mesolophid is long, reaching a point in the middle of the talonid basin. The metastylid crest is short and does not reach the mesostylid. Both mesoconid and ectomesolophid are strong; the ectomesolophid has a triangular shape. A strong oblique entolophid connects directly the entoconid to the mesoconid. The entoconulid is absent, whereas the hypoconulid is strongly developed. There is no connection between the hypoconulid and the entoconid so the posterior sinusid is open postero-lingually. The roots are not visible.

**m3:** Only one, strongly worn m3 has been found; it is longer than the m2. Only a few morphologic features are observable such as the strong and triangular ectomesolophid, the strong hypoconulid, the posterior sinusid open postero-lingually, the oblique entolophid and the talonid basin open lingually. The roots are not visible.

**Comparisons and discussion:** The molars of the mandible NHMW 2015/0375/0001 are too worn to precisely observe the morphology. However, their size and shape are similar to the mandible NHMW 2015/0373/0001; furthermore, they share a number of morphological features: a strong, triangular ectomesolophid; the posterior sinusid open postero-lingually; and the oblique entolophid. Both specimens are consequently referred to the same taxon, but the upper teeth of this taxon remain so far unknown.

Our specimens differ from the genus *Proansomys* Bi et al., 2013 by its well-developed mesostylid on lower molars, its complete entolophid on p4 and its strongly lophate metaconid in molars. They also differ from *Ninamys* by the absence of metalophule II and by the well-developed mesolophid. They differ from *Promensiscomys* by the lophate metaconid, absent metalophule II, large triangular ectomesolophid and absent hypoconid ridge. They differ from *Prosciurus* by the complete entolophid and absent metalophid II.

The teeth here studied present some similarities with *Ansomys* Qiu, 1987, but differ in being less lophodont and displaying a different shape: on the Mongolian material, the outlines are triangular because the talonid is noticeably wider than the trigonid, whereas in *Ansomys*, outlines are more squared because the anterior and posterior widths are similar. Among late Oligocene and early Miocene taxa, our specimens differ from *Ansomys shantungensis* by their larger size and absent metalophid II. They also differ from *Ansomys orientalis* by their absence of accessory crests of the trigonid basin; their shorter mesostylid disconnected from the metastylid crest.

Our specimens display a number of features that are diagnostic for the subfamily Ansomyinae as defined by Qiu (1987) and later revised by Hopkins (2008) such as the following: the brachydont cheek teeth with the absence of anteroconid crest, the metaconid reduced to a crest, the well-developed hypoconid, large mesoconid labially located and the entoconid placed anteriorly to the hypoconid. Furthermore, the Mongolian m1/2 have no metalophulid II and display a metaconid ridge that extends posteriorly (as in most members of the clade; Hopkins 2008) but disconnected from mesostylid, the condition of which is not described in any other genus of the Ansomyinae. Therefore, our two specimens might represent a new genus, possibly closely related to *Ansomys*. However, given the scarcity of material and the absence of information from the upper teeth, we tentatively refer them to the subfamily Ansomyinae Qiu, 1987, but we leave the nomenclature open until more material is found.

Genus *Proansomys* Bi et al., 2013

**Type species**: *Proansomys dureensis* Bi et al., 2013

**Other referred species**: *Prosciurus daxnerae* Lopatin, 2000 from Kazakhstan, North Aral region, Altynshokysu locality, Bone Bed 2 (Late Oligocene)

**Discussion**: Lopatin (2000) described a new species of Aplodontidae on the basis of a single lower molar from the late Oligocene of the Aral region (see Bendukidze et al. 2009 for a revision of the age of the locality). This new species was initially named as *Prosciurus daxnerae* Lopatin 2000. Later, Vianey-Liaud et al. (2013) assigned it to the newly created genus *Ninamys*. The morphology of the m1 described by Lopatin (2000, 2004) includes features which differ from the genus *Ninamys* but fit the morphology of *Proansomys* such as large and triangular-shaped ectomesolophid, short metalophid II ending free in the trigonid basin and four roots. Based on these observations, the referral of *Prosciurus daxnerae* Lopatin 2000 to *Proansomys* seems more likely. Also, the molar described by Lopatin (2000, 2004) is noticeably smaller and proportionally shorter (1.35 × 1.75 mm) than the type material of *Proansomys dureensis* (mean values 1.95 × 1.92 mm for m1, 2.00 × 2.00 mm for m2, Bi et al. 2013). This indicates that the Kazakhstan material belongs to a different species, *Proansomys daxnerae*.

*Proansomys badamae* sp. nov.

Figs. 6d and 8b–l

**Derivatio nominis**: In honor of Dr. Demchig Badamgarav† (Mongolian Academy of Sciences), Mongolian geologist and for almost 20 years guide and collaborator of the joint Austrian-Mongolian expeditions/field campaigns.

**Type locality**: Taatsiin Gol right (TGR), Uvurkhangai, Mongolia; fossil layers TGR-C/1, TGR-C/2 and TGR-C/7, red-brown claystone alternating with red-rose caliche layers, Hsanda Gol Fm., Late Oligocene (biozone C).

**Holotype**: Right mandible with p4-m3 (NHMW 2015/0374/0002; Figs. 6d and 8l) from TGR-C/1. Measurements: p4: L = 1.98, TrW = 1.43, TaW = 1.92; m1: L = 1.88, TrW = 1.49, TaW = 1.86; m2: L = 2.05, TrW = 1.70, TaW = 1.92; m3: L = 2.03, TrW = 1.60, TaW = 1.50.

**Paratypes**: TGR-C/1: 1 p4 (NHMW 2015/0374/0001); TGR-C/2: 1 M1/2, 2 fragmentary M3, 1 p4, 1 m1, 1 fragmentary m1/2 (NHMW 2015/0376/0001-0005); TGR-C/7: 1 P4, 4 M1/2, 1 M3, 1 p4, 1 m3 (NHMW 2015/0381/0001-0008).

**Diagnosis**: Large aplodontid with brachydont teeth and without accessory lophules. In upper molars, the P4 is wide with a narrow anterior lobe; on P4-M1/2, the ectoloph has a bucket-handle shape and the hypocone is either absent or barely visible. Lower molars have a short metalophid II, a well-developed metastylid crest, an antero-posteriorly compressed metaconid and the hypolophid attached to the hypoconulid rather than the ectolophid on p4, but not the entoconulid crest. There are three roots on all upper cheek teeth, two or three roots on p4 and three or four roots on upper molars. The mandible is slender, characterised by a diastema strongly curved caudally but not rostrally and a mental foramen dorsally located so it is visible in occlusal view.

**Differential diagnosis**: The new species differs from *Proansomys dureensis* in having a shorter metalophid II, a better developed metastylid crest, a wider P4, a much better developed protocone in upper molars and in missing the entoconulid crest. It also differs from *Proansomys dureensis* in displaying a noticeable variability in the development of the mesotyle, ectoloph and ectomesolophid. The new species also differs from *Proansomys daxnerae* in being noticeably larger and proportionally longer.

**Other localities and stratigraphical distribution:** Taatsiin Gol right (layers TGR-C/1, TGR-C/2 and TGR-C/7, biozone C; late Oligocene); Toglorhoi (layers TGW-A, TGW-A/2a and TGW-A/2b, biozone C; layer TGW-A/5, biozone C1; late Oligocene); Huch Teeg (layer RHN-A/7, biozone C1; late Oligocene).

**Material referred:** 21 isolated upper molars, 11 isolated lower molars and 2 mandibles; see appendix (ESM) for details.

**Measurement**: Given in Table 5, see appendix (ESM) for details.

**Table 5:** Measurements (in mm) of *Proansomys badamae* sp. nov. from the localities: Taatsiin Gol right (TGR) and Toglorhoi (TGW), local biozone C; Taatsiin Gol right (TGR), local biozone C or C1; Huch Teeg (RHN) and Toglorhoi (TGW), local biozone C1

	L						W/TaW						TrW					
	*N*	Min	Max	Mean	*σ*	CV	*N*	Min	Max	Mean	*σ*	CV	*N*	Min	Max	Mean	*σ*	CV
Biozone C-C1																		
P4	2	2.22	2.37	2.30	–	–	2	2.44	2.53	2.49	–	–		–	–	–	–	–
M1/2	7	1.57	1.79	1.71	0.07	4.29	7	2.38	2.53	2.45	0.05	2.23		–	–	–	–	–
M3	2	1.86	1.97	1.92	–	–	3	2.11	2.12	2.12	–	–		–	–	–	–	–
p4	6	1.88	2.19	2.01	0.10	5.10	6	1.79	2.14	1.93	0.13	6.92	6	1.17	1.51	1.39	0.13	9.18
m1/2	5	1.88	2.08	1.99	0.08	4.17	5	1.81	2.09	1.93	0.11	5.64	5	1.30	1.77	1.59	0.19	12.19
m3	2	2.03	2.24	2.14	–	–	2	1.50	1.72	1.61	–	–	2	1.60	1.73	1.67	–	–

*N* number of teeth measured, *Min* minimum, *Max* maximum, *σ* standard deviation, *CV* variation coefficient of Simpson

### Description

**P4:** The protocone, paracone and metacone are well-developed, whereas the hypocone is absent. The tooth is characterised by a long, narrow anterior lobe. The anterocone is large, whereas the parastyle and anterostyle are small. The ectoloph displays a characteristic bucket-handle shape but is interrupted in one specimen between the paracone and metacone; it is also less developed compared to the M1/2. The protoloph is curved; the labial part of the protoloph is transverse, whereas the lingual part is posteriorly directed. The protoloph is connected in the posterior part of the paracone and paraconule. Both the metacone and metaconule are large and antero-posteriorly elongated. The labial metaloph is always complete and usually slightly curved between the metacone and metaconule. The lingual metaloph connects to the lingual protoloph between the paraconule and protocone. The posteroloph is complete but low; it closes the postero-labial and postero-lingual sinuses posteriorly. The tooth has three roots.

**M1/2:** The protocone, paracone and metacone are well-developed, whereas the hypocone is barely visible or absent. The anterior arm of the protocone is usually absent, except for one tooth (NHMW 2015/0381/0005). Both parastyle and anterostyle are large and clearly differentiated from the anteroloph. The ectoloph is always present and always has a bucket-handle shape; it is continuous and connects the parastyle with the posteroloph. The mesoloph is absent. The development of the ectoloph varies, being weakly marked in some specimens. The presence of a mesostyle is variable. Both lingual and labial parts of the protoloph are aligned so the protoloph is straight. The protoloph is attached to the posterior part of the paraconule and paracone. The labial metaloph is always complete and usually slightly curved between the metacone and metaconule. The lingual metaloph connects on the lingual protoloph close to the paraconule. The posteroloph is complete and well-developed; it closes the postero-labial and postero-lingual sinuses posteriorly. The tooth has three roots.

**M3:** The parastyle is well-developed and labio-lingually elongated, whereas the anterostyle is weakly developed or even absent. The anterior arm of the protocone is very short. The hypocone is well-developed but smaller than the other cusps and antero-posteriorly elongated. Both lingual and labial parts of the protoloph are aligned so the protoloph is straight. The protoloph is attached to the posterior part of the paraconule and paracone. The labial metaloph is always complete and straight between the metacone and metaconule. The lingual metaloph connects on the lingual protoloph between the paraconule and the protocone. The metaconule is antero-posteriorly elongated and almost perpendicular to the labial metaloph. The tooth has three roots.

**Mandible:** The mandible is slender, and the diastema is deep and curved. The coronoid process is not preserved, but its base shows an origin lateral to the posterior part of m3. Both the dorsal and the ventral masseter crests are weakly marked. The dorsal masseter crest joins the ventral one at the middle part of m2. The dorsal masseter crest is stronger than the ventral one, but the ventral masseter crest extends slightly further anteriorly, below the posterior part of the m1. The position of the mental foramen is high, visible in the occlusal view; it is located close to the middle of the diastema, at its lowest part.

**p4:** The metalophid I is present in the form of an anterior arm of the protoconid but is generally interrupted and does not reach the metaconid, whereas the metalophid II is complete. The anteroconulid is absent. The metastylid crest is long and closes the talonid basin. Both the mesoconid and ectomesolophid are merged and the resulting form has a large triangular outline. The ectomesolophid is rarely distinguishable from the mesoconid. The entolophid is curved and connects the entoconid to the hypoconulid. The entoconulid is absent, whereas the hypoconulid is prominent. The connection between the hypoconulid and the entoconid is variable so the posterior sinusid is either open postero-lingually or closed. One tooth (NHMW 2015/0374/0001) displays an additional labial posterolophid which is short and forms a loop starting from the hypoconulid but not reaching the hypoconid. The tooth has two or three roots.

**m1/2:** The distinction between the first and second molars is not always easy based on isolated teeth. However, they usually differ by their size: the m1 is slightly smaller and noticeably shorter. Also, they differ in the entolophid: on the m2s, the entolophid always links the entoconid directly to the mesoconid, whereas on the m1s, the entolophid is variable and connects either to the mesoconid (as on m2) or to the hypoconulid (as on p4). The mesostylid and mesolophid are also usually more developed on the m2s than on the m1s. The metalophid I is connected and well-developed, whereas the metalophid II is short and ends free in the trigonid basin. The anteroconulid is weak but often visible. The mesostylid is strong and the mesolophid can be absent or short. The metastylid crest can be long or short and never reaches the mesostylid. Both mesoconid and ectomesolophid are merged and the resulting form has a large triangular outline. The ectomesolophid is rarely distinguishable from the mesoconid. The entolophid is oblique when it connects the entoconid to the mesoconid directly; it has no entoconulid or entoconulid crest so the posterior sinusid is not divided. The hypoconulid is prominent. The connection between the hypoconulid and the entoconid is variable; thus, the posterior sinusid is either open postero-lingually or closed. Concerning the roots, the m1/2s usually have three or four with different degrees of division of the anterior and posterior roots. In the cases when m1 and m2 can be clearly differentiated (two fragmentary mandibles: NHMW 2015/0378/0001 and NHMW 2015/0374/0002), the m1 has three roots and the m2 has four. By contrast, one strongly worn tooth (probably an m1: NHMW 2015/0376/0006) has only two roots. This m1 is the same size as the m1 from the mandibles but because of the wear is not possible so far to state whether this specimen actually belongs to another taxon or simply illustrates a noticeable variability on the roots of the m1s.

**m3:** The m3 is about as long as the m2 and slightly longer than the m1. The morphological features of the m3 are similar to the m2, but the talonid and trigonid widths are almost equal and it has the posterior sinusid closed. The tooth has three roots.

**Discussion:** The Mongolian material here studied is characterised by brachydont teeth without accessory lophules, ectoloph on P4-M1/2 with a bucket-handle shape, antero-posteriorly compressed metaconid, hypolophid attached to the hypoconulid rather than the ectolophid on p4 and three or four roots on m1/2. These characters fit the morphology described for the genus *Proansomys* Bi et al., 2013 that has three roots on lower molars (Bi et al. 2013). In addition, the mandible displays a diastema that differs from the above-described species in being strongly curved caudally but not rostrally (Fig. 6). For these reasons, we refer the new species to the genus *Proansomys*. The genus *Proansomys* was initially described as monospecific (Bi et al. 2013). With the new generic assignment of the species described from Altynshokysu by Lopatin (2000) and *Proansomys badamae* sp. nov., the genus *Proansomys* becomes now a relatively diverse genus characteristic of the late Oligocene of North and Central Asia. So far, the three species seem to be geographic vicariants. However, further analysis will be necessary in the future to assess the phylogenetic relationship between those species and with other Ansomyinae. More generally, the three species of *Proansomys* together with Ansomyinae indet. described above and *A. shantungensis* from the late Oligocene of the Junggar basin (Ye et al. 2003) indicate that Ansomyinae are actually, already, quite diversified in the late Oligocene of Asia. This supports the assumption previously made (Bi et al. 2013) that the origin of this clade is likely to take place somewhere in Asia during the early Oligocene, although lines of evidence are still missing in the fossil record.

Genus *Ansomys* Qiu, 1987

*Ansomys* sp.1

Fig. 9a

**Fig. 9:**
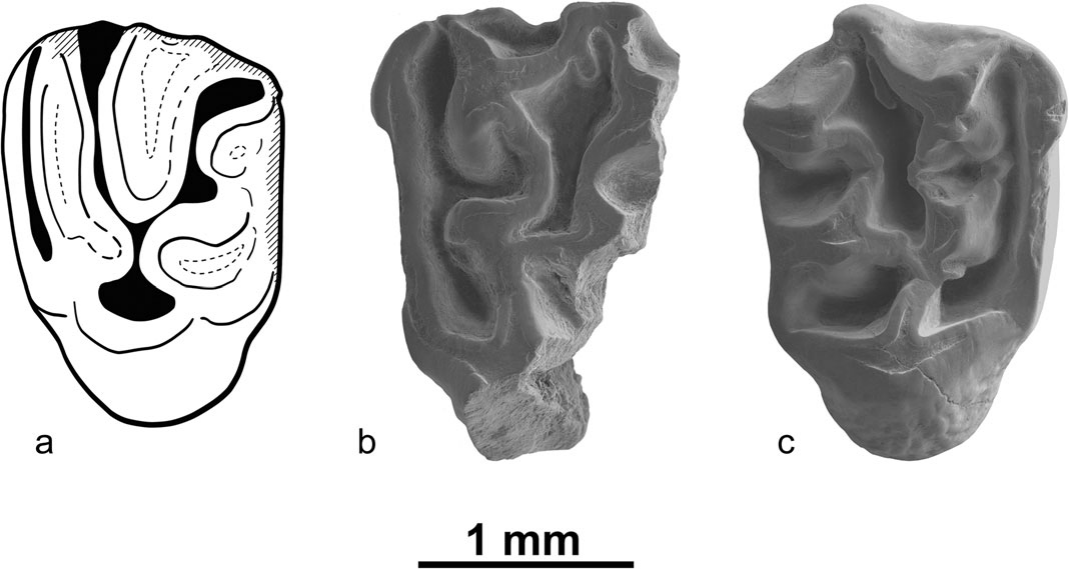
*Ansomys* sp.1 (**a**) from the early Miocene (local biozone D) of Huch Teeg (RHN) and *Ansomys* sp.2 (**b**–**c**) from the ?middle Miocene (local biozone D1/2) of Ulaan Tolgoi (UTO), occlusal views: **a** left M1/2, RHN-A/12 (NHMW 2015/0384/0001); **b** left M1/2, UTO-A/5 (NHMW 2015/0385/0002); **c** right M1/2, UTO-A/5 (NHMW 2015/0385/0001)

**Localities/stratigraphy:** Huch Teeg (layer RHN-A/12, biozone D, early Miocene).

**Material and measurements:** left M1/2 (NHMW 2015/0384/0001; 1.31 × 1.86 mm).

**Comparisons and discussion:** This molar is the only specimen of Aplodontidae found in a layer correlated to the local biozone D. The tooth is unfortunately strongly corroded, this is the reason why the measurements might be slightly underestimated and only few morphological characters can be observed. The anterior arm of the protocone merges with the anteroloph; the ectoloph seems continuous (but is partly broken); the hypocone is absent, and the metacone and metaconule are antero-posteriorly elongated. Those characters correspond to the genus *Ansomys* but do not allow a further identification of the specimen at the specific level.

*Ansomys* sp.2 (=*Ansomys* sp. sensu Qiu, 1996)

Fig. 9b, c

**Localities/stratigraphy:** Ulaan Tolgoi (layer UTO-A/5, biozone D1/2, early middle Miocene).

**Material and measurements:** Right M1/2 (NHMW 2015/0385/0001, L = 1.51, W = 2.18 mm); left M1/2 (NHMW 2015/0385/0002, fragment not measurable); left m1/2 (NHMW 2015/0385/0003, fragment not measurable).

### Description

**M1/2**: The tooth is much wider than long. The oclusal pattern is brachio-lophodont but without accessory crests. The crown is moderately high. The protocone, metacone and metaconule are lophate and antero-posteriorly elongated. The anterior arm of the protocone is merged with the anteroloph. The parastyle is strongly developed, whereas the anterostyle is absent (or not distinguishable from the anteroloph). The hypocone is absent (or not distinguishable from the posteroloph). The paracone has an angular shape producing a more or less squared wear surface. The ectoloph strongly protrudes labially forming a sharp bend (handle-shaped); on both sides labial cingula border the paracone and metacone. The mesostyle is well-developed and extends into the central sinus. The protoloph is connected to the posterior part of the paraconule and paracone. Both the labial and lingual parts of the protoloph and the labial metaloph are bent towards the centre of the tooth producing an irregular and narrow shape of the central sinus. The paraconule is small compared to the paracone; the paraconule has a short anterior arm that either ends free in the anterior sinus or forms a loop and connects to the paracone (in addition to the labial protoloph). The lingual metaloph connects on the paraconule (or the lingual extremity of the paraconule). The tooth has three roots.

**m1/2**: Represented by a single fragment, we can only observe a morphology with long, thin lophids and accessory crests.

**Comparisons and discussion:** These specimens display the diagnostic characteristics of *Ansomys* such as the lophate cusp(id)s, the handle-shape ectoloph and the trigonid basin with accessory crests. Among the species of *Ansomys* from the early Miocene of Northern Asia, it is not possible to compare with *Ansomys crucifer* Lopatin, 1997 (Northern Aral region, Kazakhstan) because it is known based on single p4. Our specimens differ from *Ansomys shanwangensis* Qiu and Sun, 1988 (Shandong, China) by the absence of a straight protoloph and split paraconule and by their smaller size. Compared to *Ansomys* sp.1 described above, the upper molars differ in being more lophate, in having a well-developed parastyle and in being larger. Our specimens fit in the lower size range of *A. orientalis*, but they differ morphologically by the absence of accessory crests on the upper molars; by their heavier built cusps and by their stronger crests. The size and morphology of our specimens seem closer to *Ansomys* sp. from Moergen V (Qiu 1996; Inner Mongolia, China). They have in common the lack (or poorly development) of accessory crests on upper molars, the absence of a hypocone, the single metaconule and paraconule, the heavier built cusps and the stronger crests. Based on those observations, we tentatively refer our specimens to the same species as in Moergen V, probably a new species but, like Qiu (1996), we prefer to leave the nomenclature open until more material is found.

## Conclusions

**Fig. 10:**
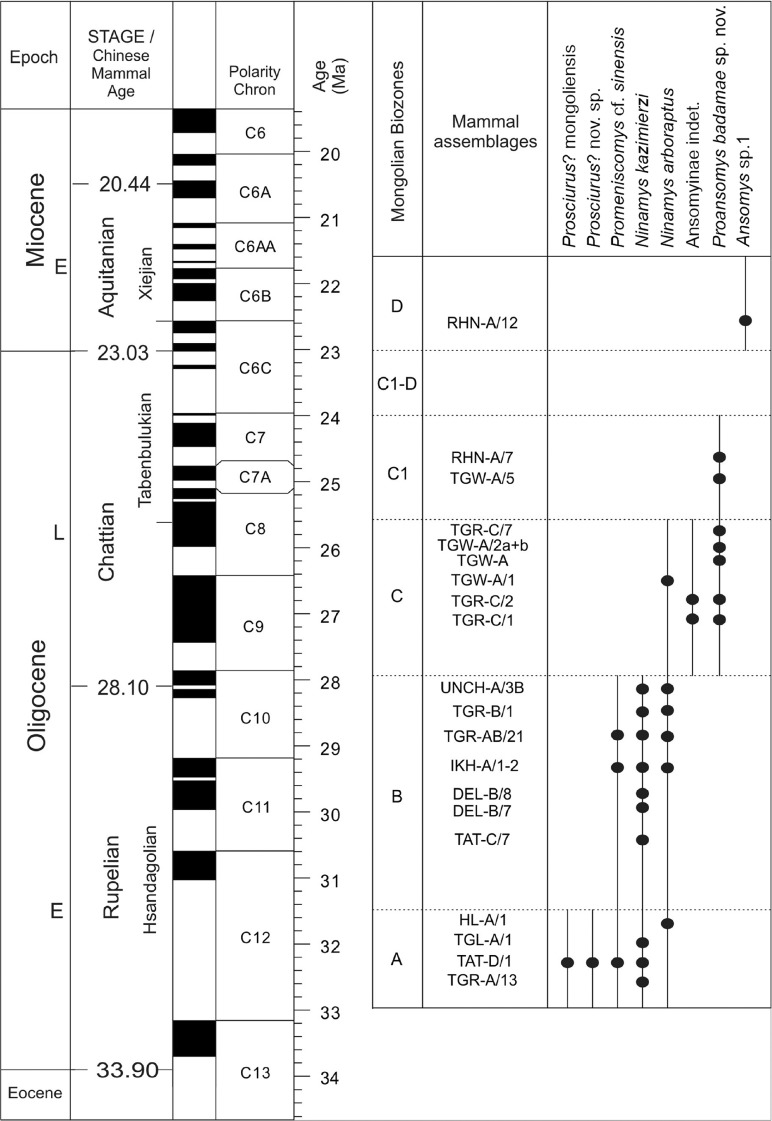
Chronostratigraphic correlation and calculation of geochronologic ages of mammal assemblages from the Valley of Lakes in Mongolia. The correlation chart includes the geologic time scale (Gradstein et al. 2012; Ogg and Lugowski 2012); the Chinese Mammal Ages Hsandagolian, Tabenbulukian and Xiejian; magnetostratigraphic data (Kratz and Geisler 2010; Sun and Windley 2015); the Mongolian biozones A, B, C, C1, C1-D and D; the respective mammal assemblages that yielded aplodontid remains; and the associated list of aplodontid taxa

Aplodontid rodents have been found in the studied area/deposits from the early Oligocene (local biozone A) to the early middle Miocene (local biozone D). Altogether, eight taxa belonging to five genera have been identified in the collection of the Austrian-Mongolian project: *N. arboraptus*, *N. kazimierzi*, *Promeniscomys* cf. *sinensis*, *Prosciurus*? *mongoliensis* and *Prosciurus*? sp. nov. in the lower Oligocene; *N. arboraptus*, *Proansomys badamae* sp. nov. and Ansomyinae indet. in the late Oligocene; and *Ansomys* sp.1 in the early Miocene (Fig. 10). The present special issue focusses on the Oligocene and early Miocene record, although some rare specimens from the ?middle Miocene (biozone D1/2) have also been found and described above: *Ansomys* sp.2 (=*Ansomys* sp. sensu Qiu 1996). Despite the fact that aplodontids are usually relatively scarce in Asian localities, 81 specimens have been recovered from the Valley of Lakes in Central Mongolia (most of the fossils from the Oligocene, only four specimens from the Miocene). For the Oligocene, the specimens illustrate a greater morphological variability than previously described. Some characters, often used as diagnostic at generic or specific levels, have, in fact, a noticeable variability (e.g. shape of the protoloph, the development of the ectoloph, the development of the hypocone, the anterior arm of the protocone, the mesostyle and the ectomesolophid). However, some species are only represented by a few specimens, and thus, the Oligocene taxa still remain poorly known. Besides, the corresponding morphology of the upper or lower cheek teeth is sometimes entirely unknown (Table 1).

Both the abundance and the taxonomic richness of aplodontids are highest in the early Oligocene and decrease through the late Oligocene. Aplodontids are rare and not very diverse in the Miocene (Table 6), their last record in the Valley of Lakes being in the middle? Miocene. This evolutionary trend contrasts to that of sciurids (Maridet et al. 2014b) which are also representatives of the Sciuromorpha and are absent from the early Oligocene, rare and not really diverse in the late Oligocene but much more diverse in the Miocene (Table 6). It has been well established for a long time (Wilson 1949), by paleontological data and numerous genetic comparisons (e.g. Huchon et al. 2002; Dawson 2003; Waddell and Shelley 2003; Fabre et al. 2012; Vianey-Liaud et al. 2013), that Aplodontidae and Sciuridae have common evolutionary origin and are close relatives. Today, both families are clearly distinguished by their skeletal adaptations, their dental patterns and their zygomatic morphology (protogomorphous vs. sciuromorphous). However, as reminded by Vianey-Liaud (1985), this morphological distinction is not so clear in the fossil record. Indeed, the oldest sciurids, like the North American late Eocene *Douglassciurus* (Emry and Korth 1996), have a protrogomorphous zygomassetic structure (Emry and Korth 1996) and both families also have very similar dental patterns up to the Miocene. This close morphologic affinity could imply that, at that time, both families had similar ecological requirements and competed for the same resources. In turn, this competition hypothesis could explain the opposite diversification trends observed in the Oligocene and Miocene fossil record of Central Mongolia. This working hypothesis, nevertheless, needs more data to be tested in the frame of a palaeoecological study.

**Table 6:**
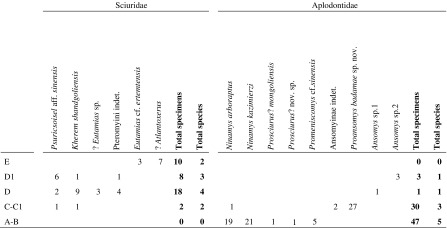
Number of specimens found in the Valley of Lakes per biozone for each species of Sciuridae (from Maridet et al. 2014b) and each species of Aplodontidae

Local biozones: A-B, early Oligocene; C-C1, late Oligocene; D, early Miocene; D1, middle? Miocene; E, late Miocene

## Electronic supplementary material

Below is the link to the electronic supplementary material.

ESM 1 (XLS 67 kb)
